# Development of a novel human CD147 knock-in NSG mouse model to test SARS-CoV-2 viral infection

**DOI:** 10.1186/s13578-022-00822-6

**Published:** 2022-06-11

**Authors:** Saiaditya Badeti, Qingkui Jiang, Alireza Naghizadeh, Hsiang-chi Tseng, Yuri Bushkin, Salvatore A. E. Marras, Annuurun Nisa, Sanjay Tyagi, Fei Chen, Peter Romanienko, Ghassan Yehia, Deborah Evans, Moises Lopez-Gonzalez, David Alland, Riccardo Russo, William Gause, Lanbo Shi, Dongfang Liu

**Affiliations:** 1 Department of Pathology, Immunology and Laboratory Medicine, Center for Immunity and Inflammation, Rutgers-New Jersey Medical School, Rutgers University, 205 S. Orange Ave., CC-H1218, Newark, NJ 07103 USA; 2 School of Graduate Studies, Biomedical and Health Sciences, Rutgers University, Newark, NJ 07103 USA; 3 Center for Immunity and Inflammation, New Jersey Medical School, Rutgers University, Newark, NJ 07103 USA; 4 Public Health Research Institute, New Jersey Medical School, Rutgers University, Newark, NJ 07103 USA; 5 Regional Bio-Containment Laboratory, Center for COVID-19 Response and Pandemic Preparedness (CCRP2), Rutgers-New Jersey Medical School, Newark, NJ 07103 USA; 6 Genome Editing Shared Resources, Cancer Institute of New Jersey, Rutgers University, New Brunswick, NJ 08901 USA

**Keywords:** CD147, Basigin, *BSG*, hCD147KI, NSG, SARS-CoV-2, COVID-19, Viral entry, Accessory receptor, Immune responses, Spike protein, Knock-in mouse model, Physiological expression

## Abstract

**Background:**

An animal model that can mimic the SARS-CoV-2 infection in humans is critical to understanding the rapidly evolving SARS-CoV-2 virus and for development of prophylactic and therapeutic strategies to combat emerging mutants. Studies show that the spike proteins of SARS-CoV and SARS-CoV-2 bind to human angiotensin-converting enzyme 2 (hACE2, a well-recognized, functional receptor for SARS-CoV and SARS-CoV-2) to mediate viral entry. Several hACE2 transgenic (hACE2Tg) mouse models are being widely used, which are clearly invaluable. However, the hACE2Tg mouse model cannot fully explain: (1) low expression of ACE2 observed in human lung and heart, but lung or heart failure occurs frequently in severe COVID-19 patients; (2) low expression of ACE2 on immune cells, but lymphocytopenia occurs frequently in COVID-19 patients; and (3) hACE2Tg mice do not mimic the natural course of SARS-CoV-2 infection in humans. Moreover, one of most outstanding features of coronavirus infection is the diversity of receptor usage, which includes the newly proposed human CD147 (hCD147) as a possible co-receptor for SARS-CoV-2 entry. It is still debatable whether CD147 can serve as a functional receptor for SARS-CoV-2 infection or entry.

**Results:**

Here we successfully generated a hCD147 knock-in mouse model (hCD147KI) in the NOD-*scid* IL2Rgamma^null^ (NSG) background. In this hCD147KI-NSG mouse model, the hCD147 genetic sequence was placed downstream of the endogenous mouse promoter for mouse CD147 (mCD147), which creates an in vivo model that may better recapitulate physiological expression of hCD147 proteins at the molecular level compared to the existing and well-studied K18-hACE2-B6 (JAX) model. In addition, the hCD147KI-NSG mouse model allows further study of SARS-CoV-2 in the immunodeficiency condition which may assist our understanding of this virus in the context of high-risk populations in immunosuppressed states. Our data show (1) the human CD147 protein is expressed in various organs (including bronchiolar epithelial cells) in hCD147KI-NSG mice by immunohistochemical staining and flow cytometry; (2) hCD147KI-NSG mice are marginally sensitive to SARS-CoV-2 infection compared to WT-NSG littermates characterized by increased viral copies by qRT-PCR and moderate body weight decline compared to baseline; (3) a significant increase in leukocytes in the lungs of hCD147KI-NSG mice, compared to infected WT-NSG mice.

**Conclusions:**

hCD147KI-NSG mice are more sensitive to COVID-19 infection compared to WT-NSG mice. The hCD147KI-NSG mouse model can serve as an additional animal model for further interrogation whether CD147 serve as an independent functional receptor or accessory receptor for SARS-CoV-2 entry and immune responses.

**Supplementary Information:**

The online version contains supplementary material available at 10.1186/s13578-022-00822-6.

## Background

SARS-CoV-2 is the novel coronavirus that causes Coronavirus-Disease of 2019 (COVID-19) and has become a global pandemic and devastated millions. While there are many similarities between SARS-CoV-2 and its sister virus SARS-CoV [1], there are many differences that have been uncovered over the past 3 years [2]. For example, several antibodies derived from convalescent patients who developed a successful immune response against the SARS-CoV virus were unable to demonstrate effective neutralization capacity against SARS-CoV-2 pseudovirus and prevent entry into target cells expressing the angiotensin converting enzyme 2 protein (ACE2) [3]. ACE2 represents the dominant entry receptor for both SARS-CoV and SARS-CoV-2 via engagement with the virion’s spike (S) protein [4]. However, it has been recently shown that due to the presence of various mutations in the receptor binding domain (RBD) domain of SARS-CoV-2, its ability to bind to ACE2 can be dramatically increased thus potentially increasing viral entry [5], escaping of antibody responses [6], and propagation of new variants through populations [7]. It has been also demonstrated that a proteolytic receptor called transmembrane protease serine 2 (TMPRSS2) also plays a significant role in priming the SARS-CoV-2 spike protein and facilitating membrane fusion [8, 9]. Recently, CD26 [10] and Neuropilin-1 [11] have also been propounded as proteins that facilitate viral entry. Another receptor called Basigin, also known as CD147, has been recently proposed to serve as an additional entry receptor for SARS-CoV-2 [12], although it is still debatable [13].

While the fundamental mechanism by which SARS-CoV-2 interacts with CD147 in humans is debated in the scientific community [13], there is a lot of potential rationales supporting the theory that CD147 could still play a role in the COVID-19 clinical course, even if it is not a functional viral entry receptor. For example, compared to ACE2 protein expression, CD147 is expressed in cardiomyocytes and endothelial cells, which may correlate with massive hemodynamic instability and cardiovascular abnormalities during SARS-CoV-2 infection [14, 15]. The high expression of CD147 protein, but not ACE2 protein, on erythrocytes and platelets, may also contribute to a "catch and clump” mechanism that increases the risk of thrombosis in various organ systems during SARS-CoV-2 infection [16]. Next, CD147 has been shown to serve as the potential entry receptor for a variety of other viral and non-viral pathogens as well, including rhinovirus [17], measles [18], meningitis [19], HIV-1 [19], and malaria [20]. This is potentially one reason that drugs such as hydroxychloroquine and azithromycin, which decrease the entry of *Plasmodium falciparum* [21–23], and Meplazumab (NCT04586153), a humanized anti-CD147 antibody, may have shown efficacy in small clinical studies during the early days of the pandemic [12, 24, 25]. However, some of these studies present several methodological weaknesses [26]. Indeed, some studies have shown that upon anti-CD147 antibody blocking, SARS-CoV-2 infection [27] and pseudotyped SARS-CoV-2 virus entry [28] are reduced, but other studies did not recapitulate this finding in other cell lines further indicating some biological nuance regarding its function [29]. However, because CD147 is implicated in many physiological [30] and immune processes [31, 32], a number of indirect mechanisms not related to viral entry may be able to explain these positive finding and provide support for further study in COVID-19 [33, 34]. Interestingly, one study observed that upon CD147 knockdown in the lung adenocarcinoma cell line CaLu3, ACE2 protein expression, but not ACE2 mRNA levels was markedly reduced [29]. Another study suggests that cell–cell transmission of SARS-CoV-2 virions may be mediated by other host receptors in addition to ACE2 further supporting the hypothesis of virion receptor promiscuity [35].

To this aim, we generated a humanized CD147 knock-in (hCD147KI) mouse model in the immunocompromised NOD-*scid* IL2Rgamma^null^ (NSG) background, which lacks a functional immune system (characterized by a lack of functional T, B, and Natural Killer cells). We performed several assays to determine whether the expression of human CD147 could sufficiently and independently predispose NSG mice to clinical manifestations of severe COVID-19 disease and found not only a moderate body weight decline in mice carrying human CD147, but also infected cells containing SARS-CoV-2 RNA 7 days post-infection. Thus, the immunodeficient background of hCD147KI-NSG mice provides a useful platform to dissect the role of specific immune cells during the SARS-CoV-2 clinical course development. Further studies using this mouse model would be able to determine if CD147 mediates viral replication in various other organs (such as the brain, gastrointestinal tract, reproductive organs, etc.) without the confounding presence of a competent host immune system.

## Materials and methods

### Genotyping for hCD147KI-NSG and hACE2KI-NSG mice

DNA from ear snips or toes was extracted using the HOTSHOT method for DNA preparation for screening mice. Briefly, mice tissues were placed in 50 µL of alkaline lysis solution (25 mM NaOH, 0.2 mM EDTA) and heated at 94 °C for 30 min. 50 µL of neutralization solution (40 mM Tris–HCl, pH 5.0) was then added to the extracted DNA. To screen mice for the hCD147 cDNA fragment, internal primers (hCD147A and B) were used to generate a 223-base pair (bp) PCR fragment. To screen mice for the hACE2 cDNA fragment, internal primers (hACE2A and B) were used to generate a 312-bp PCR fragment. To confirm proper CRISPR-guided integration of the entire hCD147 and hACE2 construct within the mouse *Bsg* and *Ace2* alleles, respectively, DNA from toe or tail biopsies were extracted using Qiagen QIAmp DNA mini kit (Qiagen, #51,306) and flanking primers were used to generate long PCR products encompassing the full length of the cDNA constructs into the targeted alleles. A complete list of primer sequences used for genotyping is provided in Additional file 1: Table S1.

### Immunohistochemistry (IHC) for CD147 on formalin-fixed paraffin-embedded (FFPE) slides

Immunohistochemistry (IHC) was performed on FFPE tissue slides following a 45-min antigen retrieval step (pH 6.0). To stain human CD147 (hCD147) and SARS-CoV-2 Spike protein RBD, the Dako EnVision + System- HRP Labelled Polymer kit (Agilent) was used according to manufacturer’s instructions against slides incubated with primary mouse anti-human CD147 antibody (Biolegend, HIM6; 1:500) or primary rabbit anti-RBD antibody (Sino Biological; 40,592-T62; 1:800), respectively. To stain mouse CD147 (mCD147), polyclonal donkey anti-goat IgG secondary (Jackson ImmunoResearch; 705–035-003 [HRP]; 1:500) was used against primary goat anti-mouse CD147 antibody (R&D Systems; BAF772; 1:100). Slides were then incubated with 3,3′-Diaminobenzidine (DAB) for 4 min before washing and counterstained with Gill No. 2 hematoxylin. Slides were then dehydrated and mounted for imaging.

### Single-cell isolation of organ tissue and flow cytometry staining

Organs and peripheral blood were obtained from euthanized adult hCD147KI^het^-NSG and WT-NSG mice. Organs were then mechanically and chemically digested with Collagenase IV (Gibco, 17,104,019) for 10 min using the gentleMACS Octo with heater (Miltenyi) before triturating through a 70 µm cell strainer. The strained fraction was centrifuged at 400 g for 5 min and then resuspended in ACK Lysis Buffer (Gibco, A1049201) for 5 min on ice. Phosphate-buffered saline (PBS) was added to quench the reaction and the cell suspension was centrifuged again. The supernatant was discarded, and the cell pellet was divided into various sample groups. Mouse cells were first preincubated with Fc Block according to manufacturer’s recommendations before proceeding to antibody incubation. Mouse CD147 was stained using primary goat anti-mouse CD147 (R&D Systems, BAF772) and visualized using Cy5-conjugated polyclonal donkey anti-goat IgG secondary (Jackson ImmunoResearch; 705–175-147). Human CD147 was stained using primary FITC-conjugated mouse anti-human CD147 antibody (Invitrogen, MEM-M6/1) Antibodies were applied at a 1:100 dilution per sample for 30 min on ice, rinsed with PBS, and resuspended in PBS. Acquisition was performed on a BD Accuri™ C6 Plus system and downstream analysis was done using FlowJo (Tree star).

### Propagation of SARS-CoV-2 virus for in vivo infections

To generate SARS-CoV-2 viral particles Vero E6 (ATCC# CRL-1586) cell monolayers were maintained in DMEM (Sigma-Aldrich; D5671) supplemented with 10% heat inactivated fetal bovine serum (Cytiva; sh30910.03). Flasks were inoculated with a dilution of 1:100 of SARS-CoV-2 USA-WA1/2020 isolate provided by the World Reference Center for Emerging Viruses and Arboviruses (WRCEVA) UTMB Arbovirus Reference Collection. After incubation at 37 °C for 1 h with gentle shaking every 15 min, inoculum was removed and replaced with fresh DMEM with 2% FBS. Stock SARS-CoV-2 virus was harvested at 72 h post-infection and supernatants were collected, clarified, aliquoted, and stored at − 80 °C until use. The virus was titrated by the standard plaque-forming assay technique and TCID/50 using VERO E6 cells as described elsewhere [36].

### SARS-CoV-2 infection in NSG, hCD147-NSG, and hACE2-NSG mice

12- to 20-week-old hCD147KI^het^-NSG and WT littermates, and hACE2KI^homo^-NSG mice were intranasally infected with 1 × 10^5^ plaque-forming units (PFU) of SARS-CoV-2 and monitored daily for clinical deterioration and body weight drop for 2 or 7 days. Similarly, age-matched WT-NSG mice were given vehicle control (DMEM). At day 2 or day 7, mice were euthanized, and lung tissues were harvested. One lobe of the lung was disrupted in 0.75 mL Trizol in a Beadbeater (VWR, Radnor, PA) for RNA analysis by qRT-PCR. One lobe of the lung was homogenized in fresh DMEM with 2% FBS and used for flow cytometry analysis. The rest of the lung was preserved in 4% paraformaldehyde (PFA) for histology.

### qRT-PCR quantification for SARS-CoV-2 viral copies in infected mice lung tissue

Real-time RT-PCR assays were carried out as described previously [37] in a 20-µL volume that contained 1 × TaqPath 1-step RT-qPCR Master Mix (A28521, Thermo Fisher Scientific, Waltham, MA), 100 nM CoV-N forward primer, 500 nM CoV-N reverse primer, and 250 nM CoV-N molecular beacon probe. Each reaction was initiated with 2 µL RNA template. SARS-CoV-2 N gene transcript reference standard was purchased from ATCC (Manassas, VA, Cat. # VR3276SD) and used for quantitative analysis of N gene transcript levels in infected mouse lung tissues. The PCR assays were performed in 200-µL white polypropylene PCR tubes (USA Scientific, Ocala, FL) in a CFX96 Touch real-time PCR detection system (Bio-Rad Laboratories, Hercules, CA). The thermal cycler was programmed to incubate the reaction mixtures for 10 min at 53 °C to generate cDNA, followed by 2 min at 95 °C to activate the DNA polymerase and then by 45 thermal cycles that consisted of DNA denaturation at 95 °C for 15 s and primer annealing and elongation at 58 °C for 60 s. Molecular beacon fluorescence intensity was monitored during the 58 °C annealing and chain elongation stage of each thermal cycle. An additional qRT-PCR assay for the mouse GAPDH gene was designed for normalization purposes. For the GAPDH assay, the thermal cycler was programmed to incubate the reaction mixtures for 10 min at 53 °C to generate cDNA, followed by 2 min at 95 °C to activate the DNA polymerase and then by 45 thermal cycles that consisted of DNA denaturation at 95 °C for 15 s and primer annealing and elongation at 60 °C for 50 s. SybrGreen (Thermo Fisher; S7567) fluorescence intensity was monitored during the 60 °C annealing and chain elongation stage of each thermal cycle. Primers were obtained from Integrated DNA Technologies (Coralville, IA) and the molecular beacon probe was obtained from LGC Biosearch Technologies (Petaluma, CA) and are listed in Additional file 1: Table S1.

### Single molecule fluorescence in-situ hybridization (sm-FISH) probe design

sm-FISH probe pools were generated to detect seven RNA + segments of the SARS-CoV-2 genome (NCBI reference: NC_045512.2). Each probe pool used (~ 48 oligo probes per gene, each probe 20 nucleotides in length) is listed in Additional file 1: Table S1. Probes were designed using Stellaris™ probe designer (Biosearch Technologies LGC, Petaluma, CA) and then synthesized and purchased from Biosearch Technologies (the probe sequences can be provided upon request). The 3ʹ-end of each probe was modified with an amine group and coupled to Texas Red-X (Fisher Scientific). Coupled probes were ethanol precipitated and purified on an HPLC column to isolate oligonucleotides linked to the fluorophore, as described previously [38]. The seven sets of probes were diluted at 25 ng/ul, pooled each set with equal volumes together, and used at 25 ng per hybridization reaction (50 ul).

### sm-FISH staining in SARS-CoV-2 infected mouse lungs and image acquisition

SARS-CoV-2 infected lung tissues were harvested at 2- or 7-days post-infection. Lungs were dissected and immediately fixed in 4% PFA, embedded in paraffin, and sectioned with a thickness of 0.7 µm. For sm-FISH staining, sections were deparaffinized, rehydrated, and subjected to heat-mediated antigen retrieval in 10 mM citrate buffer (0.173 g citric acid and 2.348 g sodium citrate in 100 mL RNAse free water, pH 6). Slides were then washed in hybridization wash buffer (10% formamide in 10% saline-sodium citrate buffer) for 20 min. In the meantime, a humidified chamber for hybridization was prepared and pre-warmed at 37 ºC, 30 min before hybridization. After washing, each slide was incubated with 50 µL hybridization buffer (10% formamide, 10% dextran sulfate, 2 mM vanadyl-ribonucleoside complex, 0.02% RNase-free BSA, 0.001% *Escherichia coli* tRNA) with labeled viral RNA probes covering the whole tissue section in the humidifier chamber overnight at 37 °C. After incubation, slides were washed twice in hybridization wash buffer for 10 min, received True Black (Biotium, Fremont, CA) for 1 min and washed with hybridization wash buffer twice. Slides were then mounted in Mounting Medium with DAPI (Abcam, Waltham, MA) and imaged using Zeiss Axiovert 200 M (20×, and 63 × oil immersion objectives) controlled by MetaMorph image acquisition software (Molecular Devices, San Jose, CA). For 63 × images, stacks of images of 16 layers with 0.2 µm interval at 100- to 2000-milisecond exposure times were used in each fluorescence color channel including DAPI. Whole sections of lung tissue from hCD147KI-NSG (n = 3), hACE2KI-NSG (n = 4), and WT-NSG (n = 7) mice was scanned and representative infection sites with viral RNA signals were imaged.

### Immunohistochemistry (IHC) for SARS-CoV-2 Spike Protein Receptor-Binding Domain (RBD) in mouse lung tissues

FFPE slides of infected lung tissues were deparaffinized and subjected to antigen retrieval at 90 °C for 40 min in 10 mM Citrate buffer. Slides were then incubated in blocking buffer [5% bovine serum albumin (BSA) in phosphate buffered saline (PBS)] at room temperature for 1 h before being stained with primary rabbit anti-RBD antibody (diluted 1:400 in blocking buffer) overnight at 4 °C. Then, slides were washed three times with PBS for 5 min before being stained with Alexa Fluor 488 conjugated goat anti-rabbit secondary antibody (Thermo Fisher; A-11034) at room temperature for 1 h. Slides were washed three times and then mounted using Prolong Gold Antifade with DAPI (Invitrogen; P36935) and sealed before imaging.

### Quantification of total and mean fluorescence intensity (MFI) for SARS-CoV-2 receptor binding domain (RBD) staining in lung tissues of infected mice

The16-bit and 8-bit images were acquired of stained lung tissue from infected and control mice. 20 random fields were imaged per mouse. To obtain final quantification, image processing was done with Python and NumPy library [39]. Matrix manipulation in NumPy automated the evaluation process. The total and mean fluorescence intensity was calculated by dividing the total signal intensity over the area within a region of interest (ROI). The ROI for lung bronchiolar regions and alveoli were calculated by considering a threshold that separates the ROI from the background. To define the threshold, the 8-bit image version, which provides clear segregations between foreground and background, was used. To obtain the unmodified pixel values from the microscopical images, selected pixels from the 8-bit images were matched with their corresponding 16-bit images, and values for SARS-CoV-2 Spike protein RBD staining from 16-bit images were used for calculations and statistics.

### Quantification of various immune cell populations in the lungs of SARS-CoV-2 infected mice by flow cytometry

Homogenized lungs suspensions from lungs of SARS-CoV-2 infected mice were rinsed and resuspended in PBS prior to staining with the following anti-mouse antibody cocktail for 30 min on ice: CD45-BV510 (BD; BDB563891), CD11b-FITC (BD; BDB557396), CD11C-Pe/Cy7 (BD; BDB558079), Ly6C-PerCP/Cy5.5 (BD; BDB560525), Ly6G-Alexa Fluor 700 (BD; BDB561236). Stained samples were then rinsed with PBS and resuspended in 4% PFA and allowed to fix for 72 h in accordance with biosafety requirements before analyzing using a BD LSRFortessa™ X-20 Cell Analyzer. Flow data was quantified using FlowJo (Tree Star). Bioinformatic analysis was performed using GraphPad Prism 5.0 (GraphPad).

## Results

### Generation of human CD147 knock-in and human ACE2 knock-in mouse using CRISPR/Cas9

**Fig. 1:**
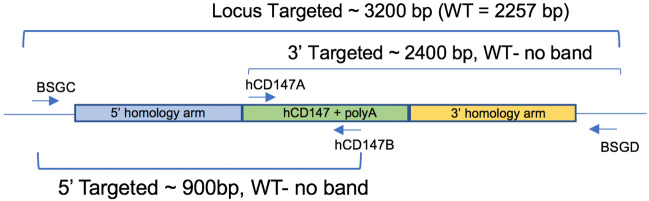
Schematic representation of genotyping primers used to confirm hCD147KI expression. A combination of 4 primers was used to screen mice (internal primers hCD147A and hCD147B) and confirm proper integration into the mouse CD147 allele (flanking primers BSGC and BSGD)

We developed a mouse model in which hCD147 was expressed in normal cells and tissue under the control of the endogenous Bsg/Cd147 gene promoter mimicking physiological expression (Fig. 1). Specifically, a human cDNA encoding CD147 was targeted to mouse CD147 exon 1 on chromosome 10. The resulting knock-in created a fusion protein with the first 22 amino acids of mouse CD147 signal peptide and amino acids 23–385 of human CD147 (NP_940991.1) that is expressed from the mouse CD147 promoter. Transcription termination was mediated by a bovine growth hormone polyadenylation signal sequence. Targeting was performed directly in NSG mouse embryos (JAX stock#: 005,557) by co-injecting a targeting vector and Cas9 protein (IDT) complexed with a CRISPR sgRNA (IDT) recognizing and cutting the sequence 5ʹ-GCCTGCGCGGCGGGTAAGAG-3ʹ. Fourteen positive founders were determined to be correctly targeted by PCR genotyping and subsequent sequencing of the targeted alleles in their entirety. Three of the 14 were determined to be biallelic at the locus. The hCD147 frequencies and antigen density are close to human CD147 expression patterns in humans [40]. A representative genotype confirms the successful generation of the hCD147KI mouse [41], and the genotyping products were verified by DNA sequencing. Female mice are fertile and were able to transmit the KI allele to offspring. However, we observed lower fertility in hCD147KI^het^-NSG males precluding the generation of a hCD147KI^homo^-NSG mouse. Thus, hCD147KI^het^-NSG mice were used for the purpose of evaluating SARS-CoV-2 sensitivity.

A similar strategy was used to generate a physiological expression model for the human angiotensin-converting enzyme 2 (hACE2) downstream of the mouse *Ace2* gene on the X chromosome in the NSG background. Human ACE2 cDNA (Genbank seqID NM_011371415.1) was inserted in frame with mouse *Ace2* using CRISPR-Cas9 in exon 1. The mouse *Ace2* 5’UTR and the first 15 amino acids of mouse *Ace2* (MSSSSWLLLSLVAVT) encoding the leader sequence were retained and the remainder of the sequence was replaced with the hACE2 cDNA sequence. Cas9 protein complexed with sgRNA containing the spacer sequence 5ʹ-GAGCAGTAGTAACAGCAACA-3ʹ and plasmid targeting vector was microinjected into NSG zygotes. Founders were screened for integration of human *ACE2* cDNA, then screened for proper targeting to the mouse locus by PCR using primers external from and internal to the targeting vector. PCR fragments were purified and sequenced by the Sanger method to confirm the cloning junctions. Founders were bred to WT NSG mice and N1 progeny were confirmed to receive the targeted allele. Thus, the hCD147KI-NSG and hACE2KI-NSG mice were successfully generated using CRISPR/Cas9 technology.

### Verification of human CD147 protein expression in various tissues by immunohistochemistry

**Fig. 2:**
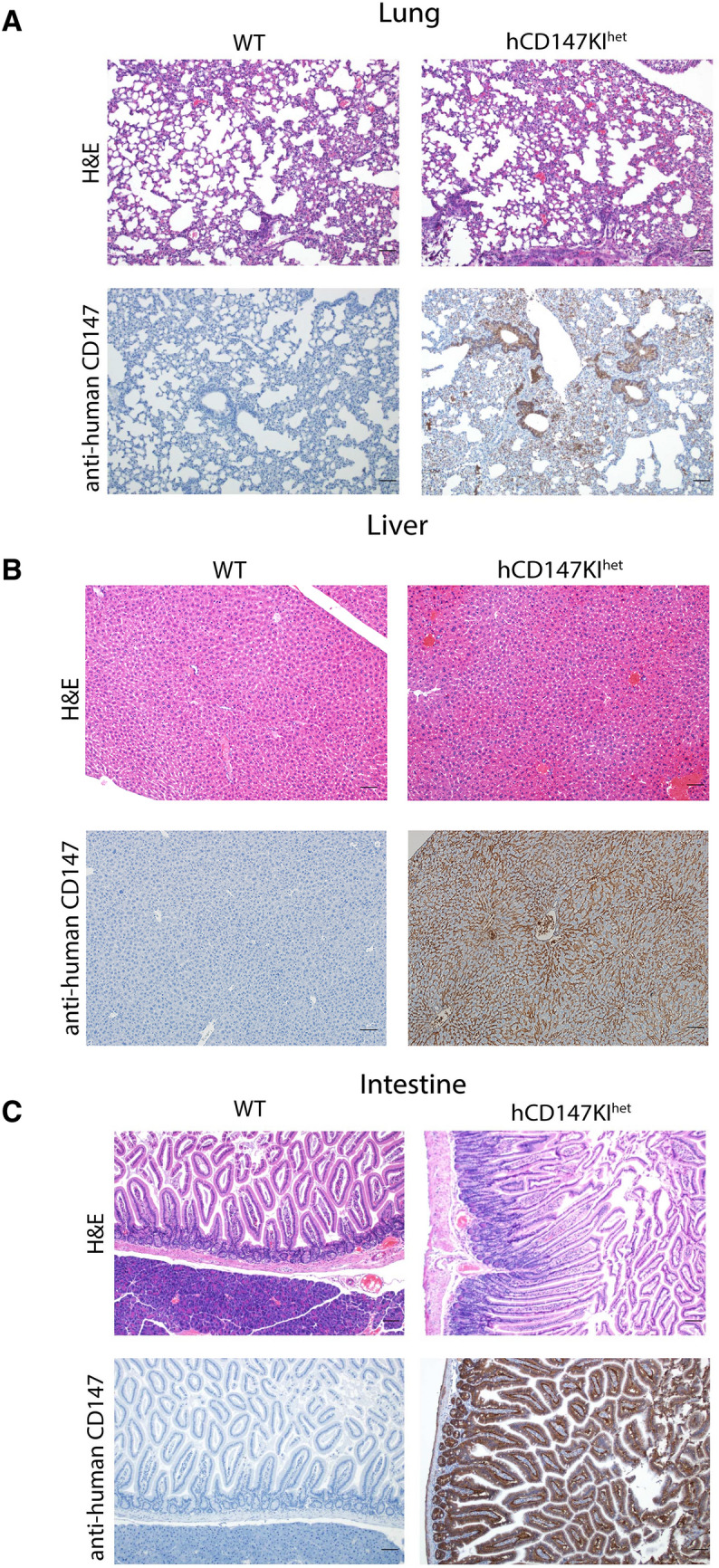

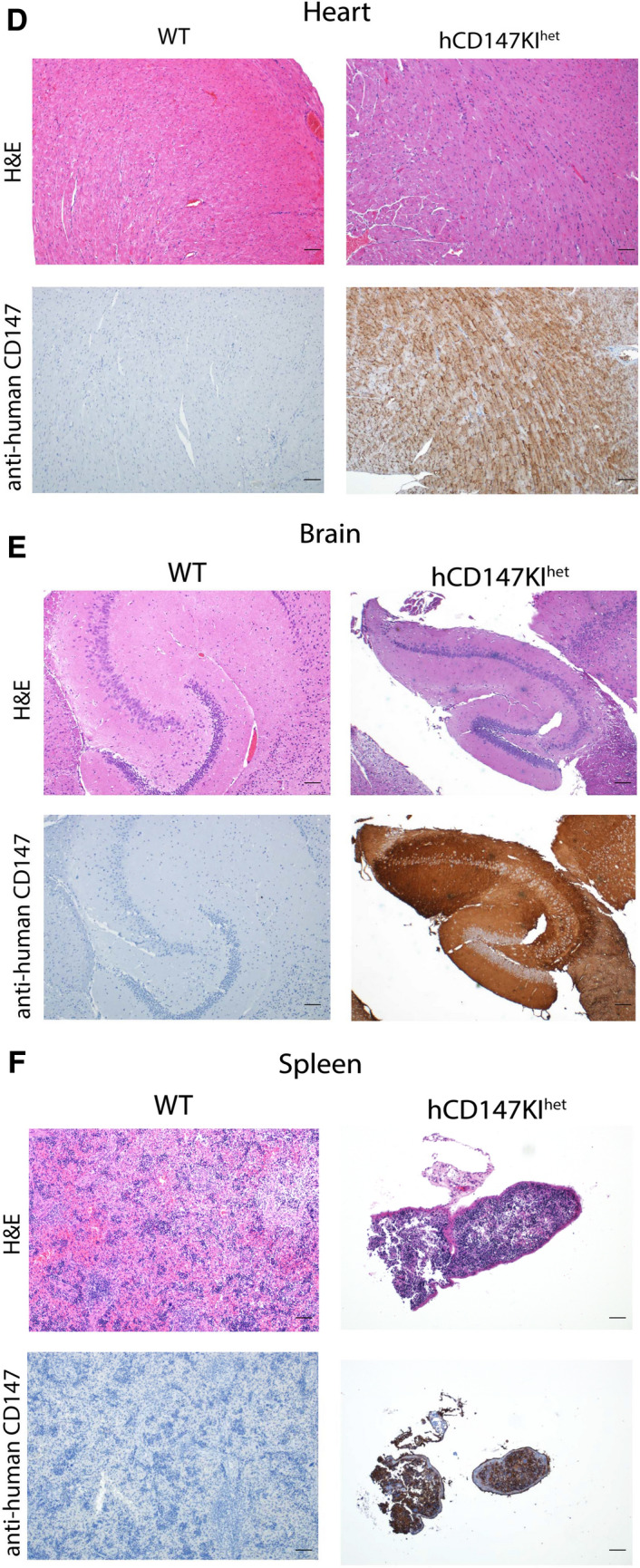

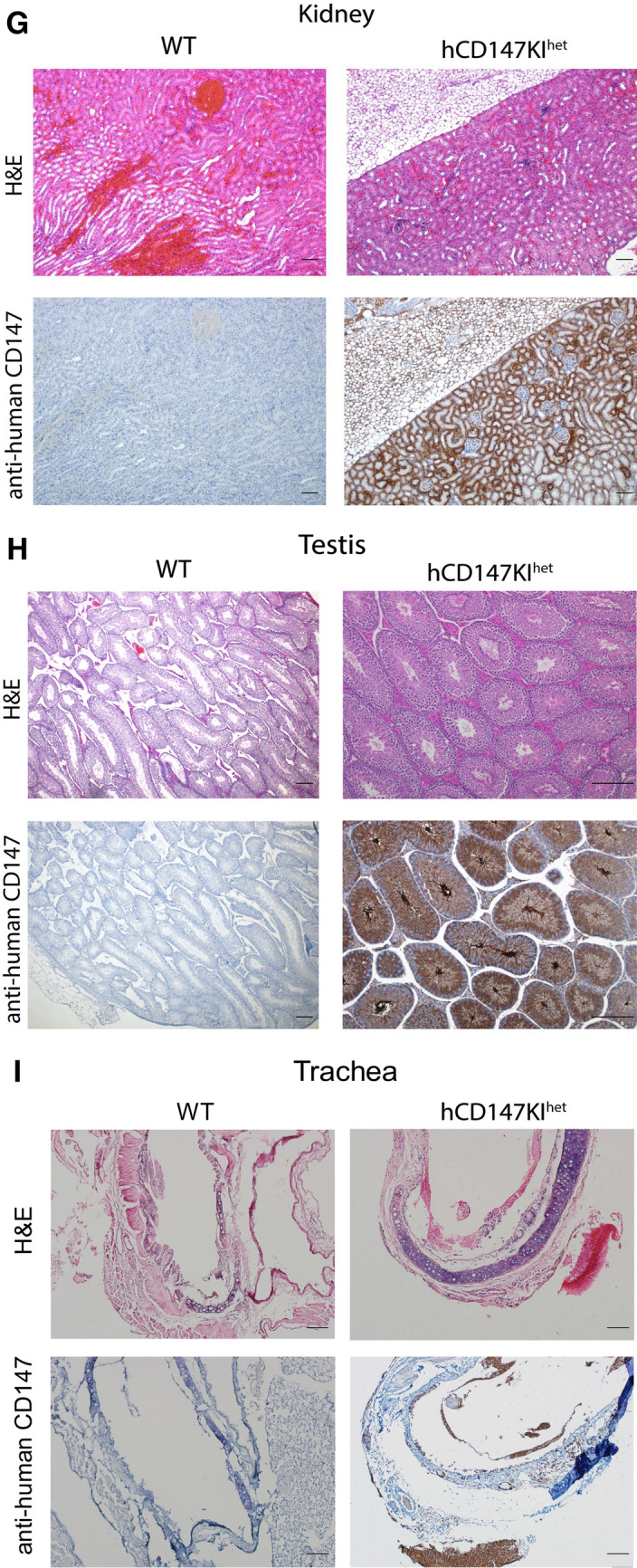
H&E and IHC of human CD147 in hCD147KI^het^-NSG mice. Human CD147 was stained (HIM6; 1:500) in the (**A**) lung, (**B**) liver, (**C**) intestine, (**D**) heart, (**E**) brain, (**F**) spleen, (**G**) kidney, (**H**) testis, and (**I**) trachea in WT-NSG (top) and hCD147KI^het^-NSG (bottom) mice. Images were taken using an Olympus Inverted Light Microscope. Scale bar represents 100 µm

After successful generation of hCD147KI^het^-NSG mice, we further verified human CD147 protein expression in these mice. Organs were harvested from adult hCD147KI^het^-NSG mice and WT-NSG littermates and stained for human CD147 protein by IHC (Fig. 2). We observed strong and specific hCD147 protein staining across all tissues assayed (lung, **2A**; liver, **2B**; intestine, **2C**; heart, **2D**; brain, **2E**; spleen, **2F**; kidney, **2G**; testis, **2H**; and trachea, **2I**) in knock-in mice compared to WT-NSG mice where no staining was visualized. Interestingly, we observed that red blood cells in the lung also expressed strong human CD147 staining indicating successful integration of the protein into erythrocyte precursors. The strongest staining was associated with bronchioles in the lungs relative to surrounding parenchyma, in condensing spermatids in the testes, and in mucosal villi in the intestines. Comparable tissue expression levels of human CD147 in hCD147KI^het^-NSG mice to that of mouse CD147 in WT-NSG mice was observed in sampled tissues as well (Additional file 1: Figure S1). Hematoxylin & Eosin (H&E) stains in WT-NSG and hCD147KI-NSG mice confirm proper architecture of each tissue (Fig. 2). Thus, we successfully generated the hCD147KI-NSG mice with human CD147 protein expression in various tissues.

### Verification of co-expression of both hCD147 and mCD147 in various tissues and blood in hCD147KI.^het^-NSG

**Fig. 3:**
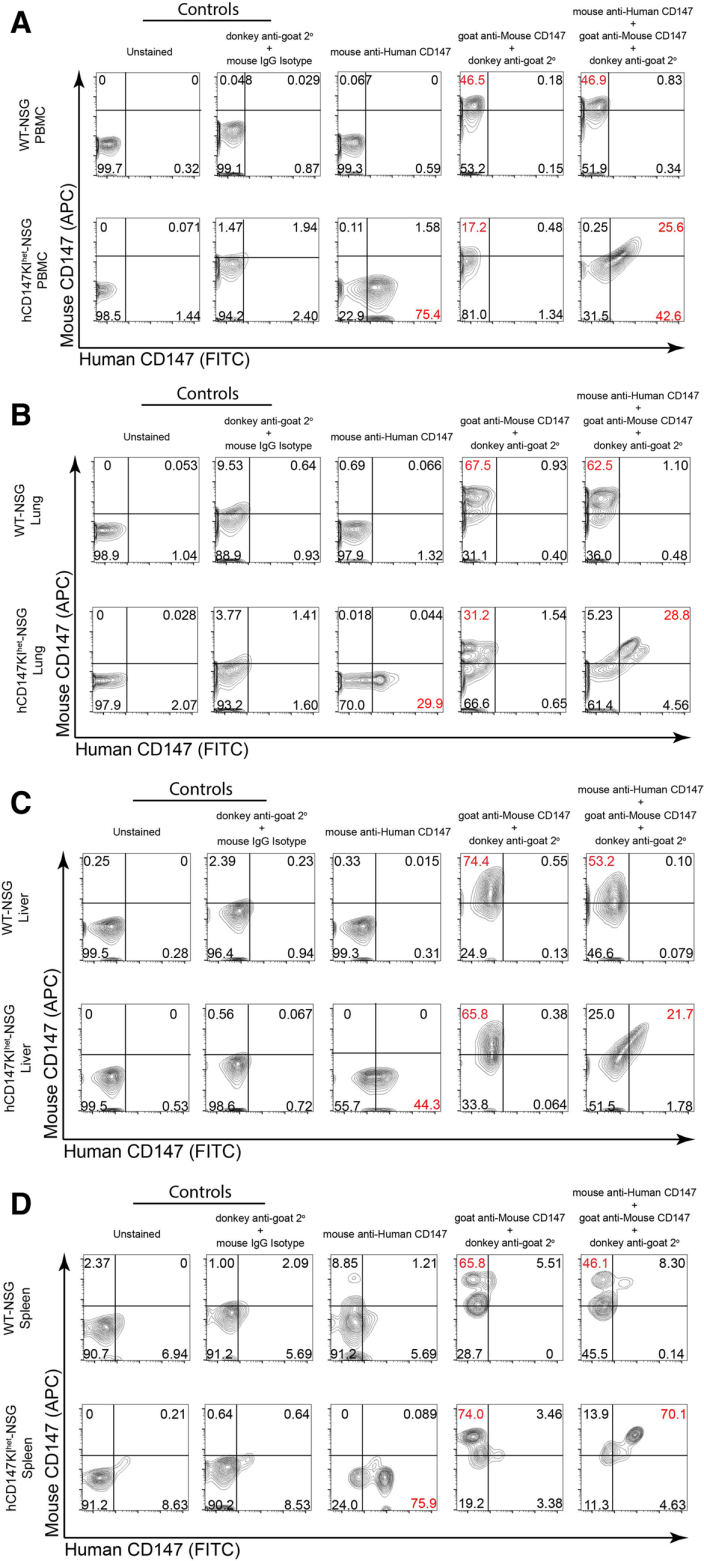
Flow cytometric analysis reveals proper dual-expression of both mCD147 and hCD147 in PBMCs and various organs. Representative contour plots of CD147 expression on WT-NSG (top) and hCD147KI^het^-NSG (bottom) cells from (**A**) PBMCs, (**B**) lung, (**C**) liver, and (**D**) spleen using antibodies targeting either mouse CD147 protein, human CD147 protein, or a combination of both antibodies (far right). Relative percentages are listed, and significant shifts highlighted in red. Gating was determined based on donkey anti-goat/mouse isotype IgG antibody background staining

To confirm proper expression of both endogenous mouse CD147 and knock-in human CD147 in hCD147KI^het^-NSG mice, we first assessed antibody specificities against CD147 to preclude any cross-reactions between human and mouse tissues by testing them against the human liver cancer cell line HepG2 and the mouse liver cancer cell line BNL 1ME A.7R.1 (Additional file 1: Figure S2). After observing no sign of cross-reaction between mouse and human cell lines, we further analyzed the expression of both proteins in various organs of hCD147KI^het^-NSG and WT-NSG mice by flow cytometry (Fig. 3). Successful co-expression of both proteins in hCD147KI^het^-NSG mice was observed in the peripheral blood mononuclear cells (Fig. 3A), lung (Fig. 3B), liver (Fig. 3C), and spleen (Fig. 3D) of knock-in mice but not in WT-NSG littermates. Together, we successfully generated the hCD147KI^het^-NSG mouse model, which can be used to test SARS-CoV-2 infection in vivo. Surface expression of the human CD147 protein was thus verified in PBMCs, Lung, Liver, and Spleen.

### Moderate sensitization of hCD147KI-NSG mice and substantial sensitivity of hACE2KI-NSG mice to SARS-CoV-2 infection, compared to WT-NSG littermates

**Fig. 4:**
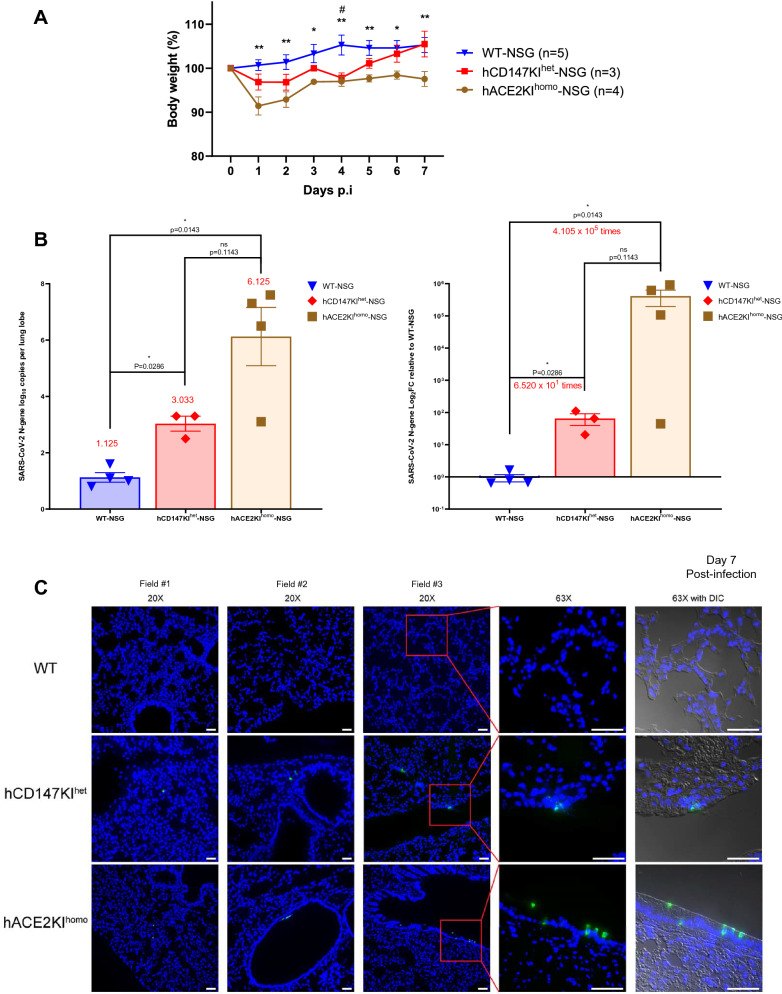
Increased sensitivity of hCD147KI-NSG and hACE2-NSG mice to SARS-CoV-2 viral infection compared to WT-NSG littermates. **A** Average body weight loss as a percent of original body weight in WT-NSG, hCD147KI^het^-NSG, and hACE2KI^homo^-NSG mice following intranasal infection with the TCID50 dose of SARS-CoV-2 virus (1 × 10^5^ PFU in 25 µL per nostril). Error bars represent standard error measure (SEM). **B** Quantification of total SARS-CoV-2 viral copies in the lungs of infected mice at day 2 post-infection by qRT-PCR, as represented by total N-gene RNA copies per lung lobe (left) and by N-gene log-twofold-change relative to WT-NSG mice (right). Mean values (red) are listed above each group. Each data point represents the average of two duplicate qRT-PCR assays from one mouse lung RNA preparation. **C** Representative sm-FISH images at 20× (first 3 columns) and 63× (4th column, zoom on region of interest along with Differential Interference Contrast (DIC) overlay) magnifications showing SARS-CoV-2 RNA detection in lung sections from WT (top), hCD147KI^het^ (middle), and hACE2KI^homo^ (bottom) mice, 7 days post-infection. Blue: DAPI; green: SARS-CoV-2 RNA. Scale bar represents 50 µm. N = 5 in WT-NSG, n = 3 in hCD147KI^het^ and n = 4 in hACE2KI^homo^ mice. Statistical significance was determined using an unpaired one-tailed t test. *indicates significance for comparisons between hACE2KI^homo^-NSG and WT-NSG mice and # indicates significance for comparisons between hCD147KI^het^-NSG and WT-NSG mice where number of symbols indicates significance strength (*or #, p < 0.05; **or ##, p < 0.01)

To evaluate whether human CD147 plays an active role in the pathogenesis of SARS-CoV-2 infection, we inoculated hCD147KI^het^-NSG mice with an infectious dose of SARS-CoV-2 (Fig. 4). We also simultaneously infected hACE2KI^homo^-NSG mice to serve as both a positive indicator for successful viral infection, as well as to evaluate any clinical deterioration in the physiologically expressing hACE2KI^homo^-NSG mice, similar to that observed in the available K18-hACE2-B6 (The Jackson Laboratory, B6.Cg-Tg(K18-ACE2)2Prlmn/J, 034,860) line. Expectedly, we observed ruffling of the fur and significant body weight drops (Fig. 4A) in hACE2KI^homo^-NSG mice at each timepoint compared to WT-NSG mice, which indicated that the method for establishing physiologically expressing mouse knock-in mice lines in the NSG background was successful. The body weights in infected hACE2KI^homo^-NSG mice did not return to initial weight levels by the end of the seven-day evaluation (Fig. 4A). Surprisingly, we observed a similar trend forming for hCD147KI^het^-NSG mice following SARS-CoV-2 infection that achieved significance at 4 days post-infection. Unexpectedly, we also observed a substantial recovery in hCD147KI^het^-NSG mice back to initial body weight levels by day seven. In a subsequent experiment, we observed slight trends in body weight drop in hCD147KI-NSG and significant body drops in hACE2KI-NSG mice and K18-hACE2KI-B6 compared to WT-NSG mice (Additional file 1: Figure S3).

To evaluate any differences in SARS-CoV-2 viral burden in the lungs of hCD147KI-NSG and hACE2KI-NSG mice, we also harvested the lungs of each mouse at day seven post-infection for immunohistochemical and molecular assessments. Consistent with previous literature [42], the hACE2KI-NSG mice developed a significant viral burden that persisted in the lungs for at least 7 days post-infection. We observed a nearly 100,000-fold increase in the presence of SARS-CoV-2 nucleocapsid RNA in the lungs of hACE2KI-NSG mice compared to WT-NSG mice by qRT-PCR (Fig. 4B). Interestingly, we also found a nearly 65-fold increase in SARS-CoV-2 viral RNA in the lungs of hCD147KI-NSG mice, compared to WT-NSG mice, supporting the hypothesis that CD147 may play an accessory role in COVID-19 disease. In a subsequent experiment where mice were sacrificed 2 days post-infection, we observed slight fold increases in SARS-CoV-2 N-gene RNA levels (Additional file 1: Figure S4) in SARS-CoV-2 infected hCD147KI-NSG mice compared to WT-NSG mice, confirming viral presence in hCD147KI-NSG mice 48 h post-infection, albeit not to the degree of that in hACE2KI-NSG mice or the significantly infected K18-hACE2-B6 transgenic mice. To confirm our observations, we performed single molecule fluorescence in situ hybridization (sm-FISH) staining on fixed lung tissues from each NSG line. Consistent with our qRT-PCR findings, we observed signs of successful infection in the lungs of both hCD147KI-NSG and hACE2KI-NSG mice (1—2 infection sites and 2—5 infection sites per examined lung section, respectively) compared to WT-NSG lungs where no infection sites were detected from multiple surveyed areas (Fig. 4C). We were also able to confirm SARS-CoV-2 viral presence in hCD147KI-NSG, hACE2KI-NSG, and K18-hACE2-B6 mice in a subsequent experiment by sm-FISH microscopy (Additional file 1: Figure S5) compared to infected WT-NSG mice 48 h after infection demonstrating higher susceptibility of these knock-in and transgenic strains to viral infection during the early infection phase. Additionally, we also observed a significant increase in total CD45^+^ lymphocytes in the lungs of all infected knock-in and transgenic lines compared to the WT-NSG mouse line 48 h after infection by flow cytometry and slight, but insignificant differences, in immune subpopulations (Additional file 1: Figure S6) relative to the parent NSG line.

To further validate the presence of viral RNA, we evaluated the presence of the SARS-CoV-2 Spike protein RBD domain by IHC (Additional file 1: Figure S7). We did observe RBD localization to the bronchiolar epithelial cells in all infected lines by conventional IHC and immunofluorescence, which is consistent with previous studies [43]. On assessing H&E sections, no significant inflammation-induced pathology in the lungs of any infected NSG mouse was observed. This is likely due to defective innate and humoral immune response systems in the NSG background. To quantify the total presence of SARS-CoV-2 in whole lung tissue histological specimens and on a per-cell basis, we calculated the mean and total fluorescence intensity of identically stained slides from infected hACE2KI-NSG, hCD147KI-NSG, and WT-NSG littermate controls (Additional file 1: Figure S8). Briefly, slides were stained for the SARS-CoV-2 Spike protein RBD domain. A total of 20 random fields of lung tissue were acquired per mouse (Additional file 1: Figure S8A). Then, image processing was performed using Python and NumPy to enumerate total signal intensity for pixels above a minimal background threshold (Additional file 1: Figure S8B). Finally, the mean fluorescent intensity per field was determined by dividing the total intensity by the total number of pixels per field above minimum threshold. Consistent with our IHC data, hACE2KI^homo^-NSG have substantially higher mean and total fluorescence intensity of SARS-CoV-2 Spike protein RBD fluorescence in lung tissues 7 days post-infection compared to control WT-NSG mice (Additional file 1: Figure S8C). In contrast, we observed no significant difference in total fluorescence between hCD147KI^het^-NSG and their WT-NSG littermates and even noticed a small, but significant decrease in the MFI of RBD protein. In conclusion, we observed an increase in SARS-CoV-2 presence in both hCD147KI-NSG and hACE2KI-NSG mice at the RNA level, and an increase in hACE2KI-NSG, but not in hCD147KI-NSG mice at the RBD protein level.

## Discussion

Whether CD147 serves as a functional receptor for SARS-CoV-2 infection is widely debated in the field of COVID-19 research. In this study, we generated two novel knock-in mouse models in the NSG background for: (1) hACE2, the putative receptor for SARS-CoV-2 entry (hACE2KI-NSG), and for (2) CD147, the potential accessory or co-receptor for coronavirus entry (hCD147KI-NSG). Next, we characterized the hCD147KI-NSG mice by flow cytometry and IHC and confirmed physiological distribution of the hCD147 protein across a variety of tissues in hCD147KI-NSG mice. Finally, we compared the ability of live SARS-CoV-2 virus to infect both knock-in models and observed significant amounts of viral RNA in lung tissues by sm-FISH and qRT-PCR.

Key to the development of successful and effective vaccines to SARS-CoV-2 infections and treatments for COVID-19 patients is the understanding SARS-CoV-2 infectivity and pathogenesis. The fundamental mechanism underlying SARS-CoV-2 entry remains poorly understood. Previous studies show that the spike proteins of SARS-CoV [4, 44] and SARS-CoV-2 [5, 45–48] bind to hACE2, a well-recognized, functional receptor that mediates viral entry. A hACE2 transgenic (hACE2Tg) mouse model is being widely used [42, 43, 49, 50], which is clearly invaluable but with some limits (e.g., low expression of hACE2 in human lung, heart, and immune cells). Other models for studying SARS-CoV-2 infection in mice are currently being optimized, including mouse-adapted virus derivations [51, 52], immunocompromised or obese mice that lack interferon receptors [52], or utilize cats, ferrets, and hamsters [53, 54].

However, while these models have recapitulated some aspects of the COVID-19 disease course, such as lung inflammation [55], cytokine storm [56], viral neuroinvasion [57], and impaired lung function [58], a majority of them cannot fully explain other aspects of COVID-19 disease, such as increased thrombosis risk, increased risk for severe COVID-19 disease in diabetic patients [59], associations between predisposing risk factors, such as stroke and immunosuppression, and clinical sequelae of COVID-19 [60–62].

Coronaviruses are known to have a high diversity of entry receptors, which includes the newly proposed human CD147 (hCD147) as a receptor for SARS-CoV-2. CD147 is a transmembrane glycoprotein with multiple functions in normal lung, immune cells, and diseased tissues [63]. It has also been shown to be expressed in the trachea [64] and in lymphoid organs such as tonsils and lymph nodes [65]. Normal epithelial and fetal tissues have low expression of CD147, when measured by immunohistochemical analysis [66]. However, CD147 is significantly upregulated in aggressive and chronic disease states, such as in cancers [67, 68], atherosclerosis [69], diabetes [70], ischemic stroke [71], and chronic lung obstruction diseases [72]. Additionally, CD147 is strongly expressed on endothelial cells in the brain [73], gastrointestinal tract tissues [74], platelets [75], conjunctival tissues [76], kidney glomerular cells and podocytes [77], and cardiac pericytes [16, 78], where it could serve a more dominant role in SARS-CoV-2 infection and mediate COVID-19-related neurological disturbance, digestive tract vascular damage, increased thrombosis, conjunctivitis, acute kidney injury, and cardiovascular disruption, respectively. Intriguingly, recent studies show that CD147 plays a functional role in facilitating SARS-CoV and SARS-CoV-2 entry [79, 80], and antibodies against CD147 block the infection capabilities of SARS-CoV-2 [12]. A humanized anti-CD147 antibody (Meplazumab) efficiently improves the recovery of COVID-19 patients with pneumonia with a favorable safety profile [81]. However, the majority of studies related to CD147, and SARS-CoV-2 are focused on cell line-based in vitro assays and protein binding experiments and have yet-to-be verified in vivo [28, 82, 83]. Additionally, recent reports found no evidence of direct interaction between CD147 protein and the RBD domain of SARS-CoV-2 [13, 84]. Thus, it is imperative to verify a potential functional role of CD147 in a live mouse model.

The hCD147KI-NSG and hACE2KI-NSG mice developed in this study utilize the natural promoter for mouse *Bsg* and *Ace2*, respectively. The human keratin 18 promoter was used to overexpress hACE2 receptor in the epithelial cells of the most commonly used K18-hACE2-B6 model. Specifically, over-expression of human ACE2 was identified in airway epithelia and the epithelia lining the liver, kidney and gastrointestinal tract in K18-hACE2-B6 mice [43]. However, expression was not observed in alveolar epithelial cells. Additionally, as multiple integrations were determined to exist on mouse chromosome 2, hemizygous mice are estimated to have between 8 full copies (or 12–30 partial copies) (commissioned analysis by The Jackson Laboratory) which is essential to establishing such a robust model for SARS-CoV-2 infection. Unlike the COVID-19 disease process in humans which has a global mortality rate of between 1 and 10% [85], a majority of SARS-CoV-2 infected K18-hACE2-B6 mice succumb to the disease process at an extremely rapid pace (as early as 6 days post-infection), making it difficult to study immunological responses [42].

CRISPR/Cas9 protein complexed with gRNA, along with the hCD147 or hACE2 plasmid constructs, were microinjected directly into NSG mouse zygotes, in order to generate a scarless knock-in of the human cDNA in frame within the first 22 amino acids of the mouse CD147 protein. Therefore, the human CD147 receptor and human ACE2 receptor are both expected to follow the same pattern of expression as the endogenous mouse receptor equivalents under normal conditions in hCD147KI-NSG and hACE2KI-NSG mice, respectively. Since the ACE2 gene is located on the X chromosome in humans and mice, we elected to utilize homozygous male and female hACE2KI-NSG mice (carrying either one or two knock-in alleles, respectively) as controls for in vivo infection experiments to eliminate the possibility for varying mouse ACE2 protein expression between homozygous hACE2KI-NSG male and heterozygous hACE2KI-NSG female mice.

**Fig. 5:**
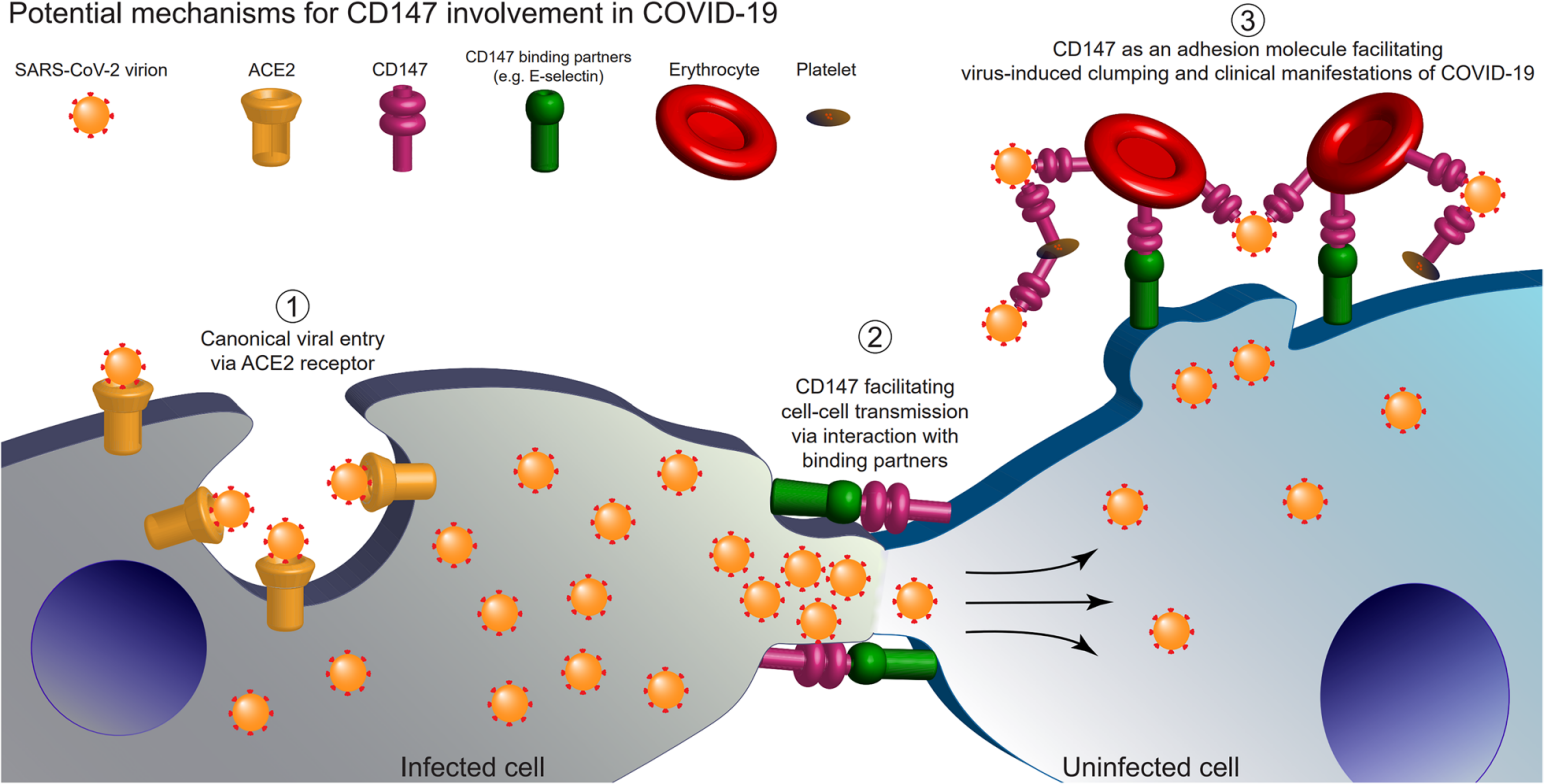
Diagram of proposed working hypothesis of CD147 in SARS-CoV-2 infection. (1) SARS-CoV-2 virions infect human cells via the canonical pathway where host Angiotensin-converting Enzyme 2 (ACE2) receptors bind to viral spike proteins (red) and facilitate viral entry and infection. (2) CD147 proteins, via binding to surface binding partners (e.g., E-selectin), facilitate cell–cell adhesion, membrane fusion, and intercellular transfer of SARS-CoV-2 virions. (3) Erythrocytes and platelets which strongly express CD147, bind SARS-CoV-2 virions, and increase thrombosis risk and other clinical manifestations of COVID-19

Our data supports the hypothesis that human CD147 plays an accessory role in SARS-CoV-2 infection. We confirmed strong expression of the human CD147 protein in the bronchiolar airway cells in hCD147KI-NSG mice compared to lung parenchymal cells. We have also confirmed expression in extra pulmonary organs such as the trachea. This protein distribution pattern may help explain why newer variants can replicate faster and more efficiently in the upper airway compared to lower airway regions [86]. Importantly, we observed increased viral presence measured by sm-FISH and qRT-PCR in the lungs of hCD147KI-NSG and hACE2KI-NSG mice, compared to WT-NSG littermates at both 2 days and 7 days after infection. While the overall degree of infection in hACE2KI-NSG mice was considerably more severe than that in hCD147KI-NSG mice, we observed a significant body weight drop, higher SARS-CoV-2 RNA level, and detected multiple SARS-CoV-2 infected cells in the lungs of both hCD147KI-NSG and hACE2KI-NSG mice for up to 7 days post-infection, compared to WT-NSG mice. We also suspect that hCD147KI-NSG and hACE2KI-NSG mice may develop increased leukocytosis in the lungs shortly after SARS-CoV-2 infection. Unexpectedly, we did not observe a similar correlation in RBD levels in hCD147KI-NSG mice. This could be explained by three possible reasons: (1) sm-FISH and IHC may have different levels of sensitivity because sm-FISH detects the RNA expression level of SARS-CoV-2, which may represent an earlier window timepoint for detection, compared with IHC detection as it detects the protein. (2) this may be due to a divergence between viral RNA replication and viral protein production/clearance (or regulation) during the recovery phase seven-day post-infection, (3) CD147 may play a role in late phase cell-to-cell infection (Fig. 5), but not as a functional receptor (such as ACE2) to mediate viral entry, evidenced by the lack of a significant difference in viral load between infected WT-NSG and in hCD147KI-NSG mice measured by qRT-PCR at the early infection (Day 2).

Additionally, CD147 is an important immune-modulator [31] making it difficult to dissect mechanisms in infection models utilizing humanized immunocompetent mice. NSG mice, on the other hand, are immunocompromised and allow for unbiased evaluation of SARS-CoV-2 infectivity without immunological interference.

The limitations of the current study include small sample sizes and lack of exact molecular mechanisms. To better assess the possibility that the human CD147 receptor contributes to overall SARS-CoV-2 infection, we are currently generating a double knock-in hCD147KI-hACE2KI-NSG mouse model to evaluate synergistic effects between the two receptors. In concordance with our previous results, we hypothesize that this mouse model will be more likely to develop significant morbidity and demonstrate a heightened sensitivity to SARS-CoV-2 infection. Additionally, future experiments will be able to compare the natural course of SARS-CoV-2 infection in immunocompromised hCD147KI-NSG and immunocompetent hCD147KI-B6 mice and will further elucidate the role of the human CD147 receptor during the SARS-CoV-2-induced immune response. While our results do not provide clarity on the mechanism by which this receptor enhances viral entry or exacerbates viral replication and persistence, we confirm here that this model can set the stage for future inquiry and examination.

The hCD147KI model offers several strengths to the scientific community as it will better capture other nuances of the COVID-19 disease. (1) This model will allow researchers to study hemodynamic instability and increased thrombosis risk following COVID-19 infection as the hCD147 protein is expressed in circulating erythrocytes. (2) The NSG background will allow scientists to study how adoptive transfer can either dampen or exacerbate COVID-19-induced cytokine storm that is often seen in severe disease. Additionally, as the NSG background has been used to study diabetes [87], our model will allow further studies into the role of diabetes in COVID-19. (3) This model can be crossed with other mouse models to determine whether a combination of human CD147 and other viral entry-related receptors (e.g., ACE2, TMPRSS2) can exacerbate clinical disease as well via interactions between the two receptors to facilitate cell-to-cell transmission in different tissues or different time-points. If such a phenomenon is observed, the exact function of CD147 as an accessory receptor to the dominant viral entry receptor, ACE2, can be interrogated and properly confirmed in vivo (Fig. 5). (4) As the human CD147 protein is expressed at physiological levels in these mice, this model will better recapitulate true physiological conditions and expression patterns normally observed in mice and humans. Even if CD147 is later determined to play a relatively minor role compared to ACE2 in SARS-CoV-2 viral entry, this mouse model may prove to be invaluable for understanding how the virus globally impacts CD147-positive cells and tissues in the in vivo setting and how therapies may modulate COVID-19 disease via this receptor. (5) These models can be used to test the infectivity and pathogenesis of the emergence of variants of SARS-CoV-2, such as B.1.1.7 [alpha], B.1.351 [beta], B.1.617.2 [delta], P.1 (20J501Y.V3) [gamma], and B.1.621 [mu], and B.1.1.529 [omicron] to additional co-receptors, given recently reported studies showing mutation-driven extension of host range to common laboratory mice [88] and increased dependence on other accessory receptors other than ACE2 [86]. (6) As CD147 is expressed on many tissues and cell types outside of the respiratory system, if in fact this receptor is implicated in viral entry and dissemination, the hCD147KI-NSG model could help shed light onto the nature of chronic COVID-19 syndrome, also known as long COVID. (7) The hCD147KI-NSG background with hACE2 mouse model can be used to test novel immunotherapies (e.g., CAR-NK) to prevent and/or treat new mutants of SARS-CoV-2 in the future. In summary, the newly generated hCD147KI-NSG mouse model can be used as a platform where direct clinical implications for vaccine and therapeutic strategies can be evaluated in preparation for future global pandemics.

## Conclusions

Currently available animal models available for the study of SARS-CoV-2 infection cannot explain many aspects of COVID-19 disease as it presents in humans such as increased thrombosis risk, increased risk of severe disease in patients with Diabetes or other comorbidities, and other clinical sequelae associated with SARS-CoV-2 infection. It has been suggested that due to its ability to serve as an entry receptor for other viruses including other coronaviruses, CD147, also known as Basigin, was recently proposed as a viral entry receptor for SARS-CoV-2. Here, we have successfully developed and validated expression of the human CD147 receptor in immunocompromised NSG mice for the purposes of evaluating whether this protein can also serve as an additional functional or accessory receptor for SARS-CoV-2 viral entry and subsequent immune responses. Our data show that similar to the newly generated hACE2KI-NSG mice, hCD147KI-NSG mice are also more sensitive to SARS-CoV-2 infection compared to WT-NSG mice characterized by a moderate body weight decline post-infection correlated with persistent viral presence in the lungs for up to 7 days post-infection. However, the effect may not be induced by direct receptor binding via CD147. The hCD147KI-NSG mouse model generated in this study will help further elucidate the molecular mechanism(s) of CD147 in SARS-CoV-2 infection.

## Supplementary Information

**Additional file 1: Figure S1.** Similar distribution of transgenic hCD147 protein by immunohistochemistry in various tissues in hCD147KI^het^-NSG mice relative to WT-NSG mice. Tissues from wild-type NSG mice (left) were stained for mouse CD147 protein using rabbit anti-mouse primary antibody (R&D Systems, BAF772; 1:100) and tissues from hCD147KI^het^-NSG mice (right) were stained for human CD147 protein using mouse anti-human primary antibody (HIM6; 1:500). Images were taken using an Olympus Inverted Light Microscope. Scale bar represents 100 µm. **Figure S2.** Specificity of anti-human CD147 and anti-mouse CD147 antibodies tested against human and mouse cell lines. Representative contour plots of CD147 expression on BNL 1ME A.7R.1 (top) and HepG2 (bottom) cells using antibodies targeting either mouse CD147 protein, human CD147 protein, or a combination of both antibodies (far right). Relative percentages are listed, and significant shifts highlighted in red. Gating was determined based on donkey anti-goat and mouse isotype IgG antibody background staining. **Figure S3.** Reduction of body weight 2 days post-infection in SARS-CoV-2 infected hACE2KI-NSG and K18-hACE2-B6 mice, and slight body weight reduction in infected hCD147KI-NSG mice. Average body weight loss as a percent of original body weight in WT-NSG (n=3), hCD147KI-NSG (n=3), hACE2KI-NSG (n=4), and K18-hACE2-B6 (n=3) mice following intranasal infection with the TCID50 dose of SARS-CoV-2 virus (1×10^5^ PFU in 25ul per nostril). Error bars represent standard error measure (SEM). Statistical significance was determined using an unpaired one-tailed Student t test. *indicates significance of p<0.05. **Figure S4.** Trends in increased viral presence 2 days post-infection in the lungs of SARS-CoV-2 infected hCD147KI-NSG, hACE2KI-NSG, and K18-hACE2-B6 mice. Quantification of total SARS-CoV-2 viral copies in the lungs of infected mice at day 2 post-infection by qRT-PCR represented as total N-gene RNA copies per lung lobe (left) and as N-gene log-2 fold-change relative to WT-NSG mice (right). Mean values (red) are listed above each group. Each data point represents the average of two duplicate qRT-PCR reactions from one mouse lung RNA preparation. Statistical significance was determined using an unpaired one-tailed t test. **Figure S5.** Increased viral presence 2 days post-infection in the lungs of SARS-CoV-2 infected hCD147KI-NSG, hACE2KI-NSG, and K18-hACE2-B6 mice by sm-FISH imaging. Representative sm-FISH images at 20X (1st column) and 63 × (2^nd^ column, zoomed on region of interest along with Differential Interference Contrast (DIC) overlay) magnifications showing SARS-CoV-2 RNA detection in lung sections from WT-NSG (1st row), hCD147KI-NSG (2nd row), hACE2KI-NSG (3rd row), and K18-hACE2-B6 (4th row) mice, 2 days post-infection. Blue: DAPI; green: SARS-CoV-2 RNA. Scale bar represents 50 µm. **Figure S6.** Increased leukocytosis 2 days post-infection in the lungs of SARS-CoV-2 infected hCD147KI-NSG, hACE2KI-NSG, and K18-hACE2-B6 mice. Quantification of various immune cell subpopulations in the lungs of SARS-CoV-2 infected mice was carried out by flow cytometry. Leukocytes were enumerated as a percentage of total live cells. Subsequent immune subpopulations are shown as percentages of total CD45^+^ cells. Error bars represent standard error measure (SEM). Statistical significance was determined using an unpaired one-tailed Student t test. * indicates significance strength where *signifies p < 0.05, **signifies p < 0.01, and ***signifies p < 0.001). **Figure S7.** H&E and immunohistochemical staining of human CD147 and SARS-CoV-2 Spike protein Receptor Binding Domain (RBD) in infected hCD147KI^het^-NSG and hACE2KI^homo^-NSG mouse lungs. Representative fields of H&E (top row), human CD147 protein (middle row), and SARS-COV-2 Spike protein RBD (bottom row) in infected WT-NSG (left column), hCD147KI^het^-NSG (middle column), and hACE2KI^homo^-NSG (right column) mice lung tissues harvested 7 days post infection. Scale bar represents 20 µm. **Figure S8.** Immunohistochemical staining and quantification of SARS-CoV-2 spike protein in lungs of infected WT-NSG, hCD147KI^het^-NSG, and hACE2KI^homo^-NSG mice. (A) Representative fields of lung tissues from infected WT-NSG (top row), hCD147KI^het^-NSG (middle row), and hACE2KI^homo^-NSG (bottom row) mice stained for SARS-CoV-2 Spike protein Receptor Binding Domain (RBD) (1st column), human CD147 (2nd column), DAPI (3rd column), differential interference contrast (DIC) (4th column), merged image (5th column). Images were acquired with an Olympus confocal microscope using a 60 × oil objective. Scale bar represents 20 µm. (B) Representative images for quantification of SARS-CoV-2 Spike protein Receptor Binding Domain (RBD) mean and total fluorescent intensity in infected lung tissues. An example of an 8-bit rescaled image (left) and its corresponding transformation after threshold cutoff was applied (right) are shown of a single field from an hACE2KI^homo^-NSG mouse lung 7 days post-infection. Scale bar represents 20 µm. (C) Relative quantification of mean and total fluorescent intensity of SARS-CoV-2 Spike protein Receptor Binding Domain (RBD) in infected lung tissues of hCD147KI^het^-NSG, and hACE2KI^homo^-NSG mice compared to control WT-NSG mice. Violin plots of mean (left) and total (right) fluorescent intensity values obtained from at least 20 individual fields per mouse lung tissue section from infected hCD147KI^het^-NSG and hACE2KI^homo^-NSG mice compared to control WT-NSG mice are shown. Statistical significance was determined using an unpaired two-tailed Student t test. The average mean (red) and total (black) fluorescence intensity value are listed beneath each violin plot. **Table S1.** Genotyping primers, RNA-FISH probes, and qRT-PCR primers and probes. A complete list of primers used to genotype hCD147KI-NSG and hACE2KI-NSG mice (top) is shown, followed by a list of the primers and molecular beacon probe used for SARS-CoV-2 quantification in infected lung tissues by Quantitative Reverse Transcription-Polymerase Chain Reaction (qRT-PCR) (bottom left) and Single Molecule RNA Fluorescent in-situ Hybridization (sm-FISH) (bottom right). CFR = Cal Fluor Red 610, BHQ-2 = Black Hole Quencher 2, underlined nucleotides in the molecular beacon probe indicate the 5ʹ and 3ʹ arm regions of the probe.

## Data Availability

The data that support the findings of this study are available from the corresponding authors upon reasonable request.

## Abbreviations

Bp: Base-pair
BSA: Bovine serum albumin
COVID-19: Coronavirus-disease of 2019
DAB: 3,3ʹ-Diaminobenzidine
FFPE: Formalin-fixed paraffin embedded
H&E: Hematoxylin & eosin
hACE2: Human angiotensin-converting enzyme 2
hACE2Tg: Human angiotensin-converting enzyme 2 transgenic
hCD147: Human CD147
hCD147KI: Human CD147 knock-in
IHC: Immunohistochemistry
K18-hACE2-B6: B6.Cg-Tg(K18-ACE2)2Prlmn/J
mCD147: Mouse CD147
MFI: Mean fluorescence intensity
NSG: NOD-scid IL2Rgammanull
PBS: Phosphate-buffered saline
PFA: Paraformaldehyde
PFU: Plaque-forming units:
RBD: Receptor binding domain
S: Spike
Sm-FISH: Single-molecule fluorecense in-situ hybridization
TMPRSS2: Transmembrane protease serine 2
