# Supplementary material for: Development of a novel human CD147 knock-in NSG mouse model to test SARS-CoV-2 viral infection
Source: Cell Biosci. 2022 Jun 11;12:88. doi: 10.1186/s13578-022-00822-6 (PMC9187929; doi:10.1186/s13578-022-00822-6)
Supplement: Supplementary file 1 — Additional file 1: Figure S1. Similar distribution of transgenic hCD147 protein by immunohistochemistry in various tissues in hCD147KIhet-NSG mice relative to WT-NSG mice. Tissues from wild-type NSG mice (left) were stained for mouse CD147 protein using rabbit anti-mouse primary antibody (R&D Systems, BAF772; 1:100) and tissues from hCD147KIhet-NSG mice (right) were stained for human CD147 protein using mouse anti-human primary antibody (HIM6; 1:500). Images were taken using an Olympus Inverted Light Microscope. Scale bar represents 100 µm. Figure S2. Specificity of anti-human CD147 and anti-mouse CD147 antibodies tested against human and mouse cell lines. Representative contour plots of CD147 expression on BNL 1ME A.7R.1 (top) and HepG2 (bottom) cells using antibodies targeting either mouse CD147 protein, human CD147 protein, or a combination of both antibodies (far right). Relative percentages are listed, and significant shifts highlighted in red. Gating was determined based on donkey anti-goat and mouse isotype IgG antibody background staining. Figure S3. Reduction of body weight 2 days post-infection in SARS-CoV-2 infected hACE2KI-NSG and K18-hACE2-B6 mice, and slight body weight reduction in infected hCD147KI-NSG mice. Average body weight loss as a percent of original body weight in WT-NSG (n=3), hCD147KI-NSG (n=3), hACE2KI-NSG (n=4), and K18-hACE2-B6 (n=3) mice following intranasal infection with the TCID50 dose of SARS-CoV-2 virus (1×10^5^ PFU in 25ul per nostril). Error bars represent standard error measure (SEM). Statistical significance was determined using an unpaired one-tailed Student t test. *indicates significance of p<0.05. Figure S4. Trends in increased viral presence 2 days post-infection in the lungs of SARS-CoV-2 infected hCD147KI-NSG, hACE2KI-NSG, and K18-hACE2-B6 mice. Quantification of total SARS-CoV-2 viral copies in the lungs of infected mice at day 2 post-infection by qRT-PCR represented as total N-gene RNA copies per lung lobe (left) and as [file 13578_2022_822_MOESM1_ESM.pptx]

## Slide 1
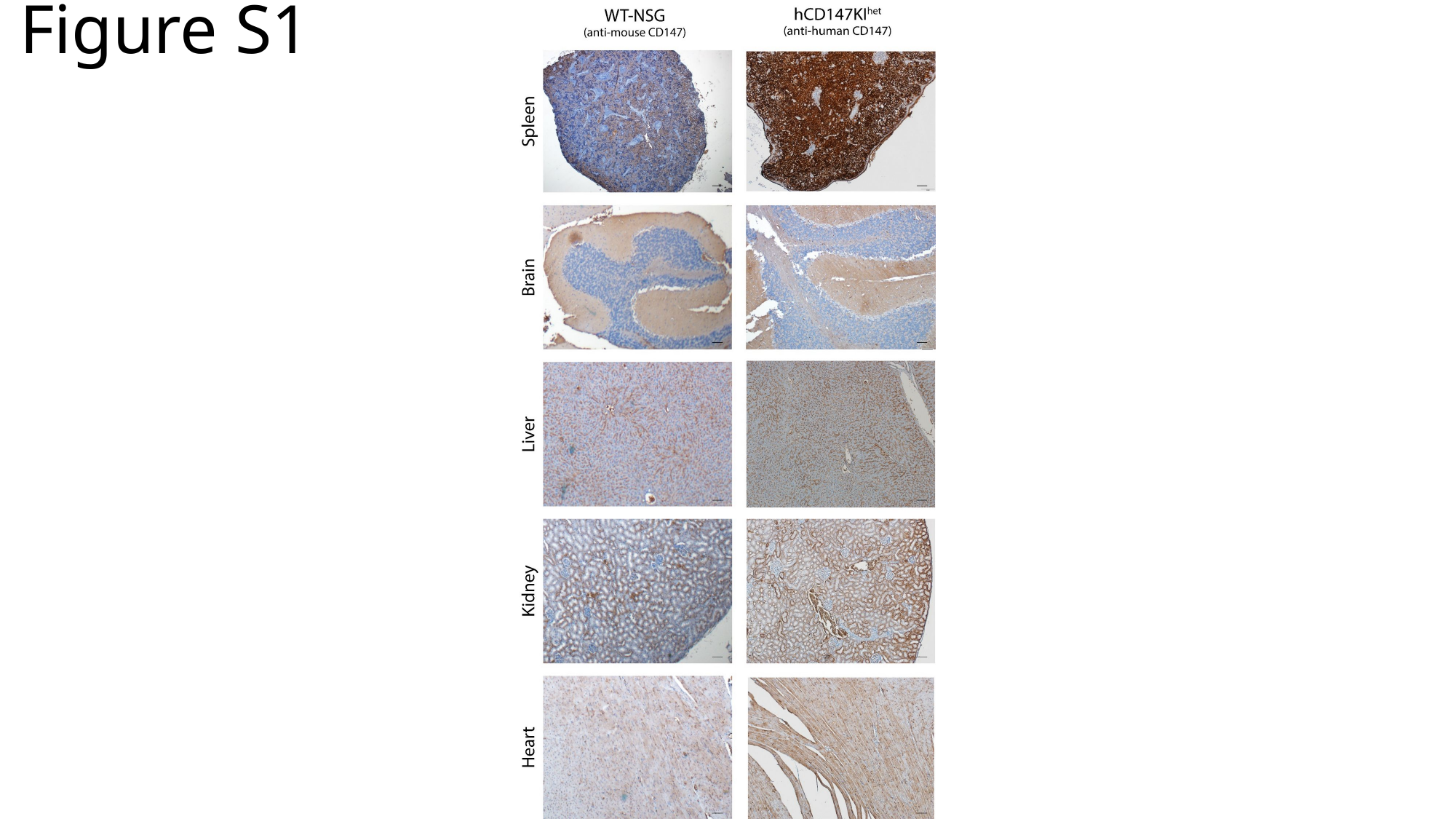

# Figure S1

## Slide 2
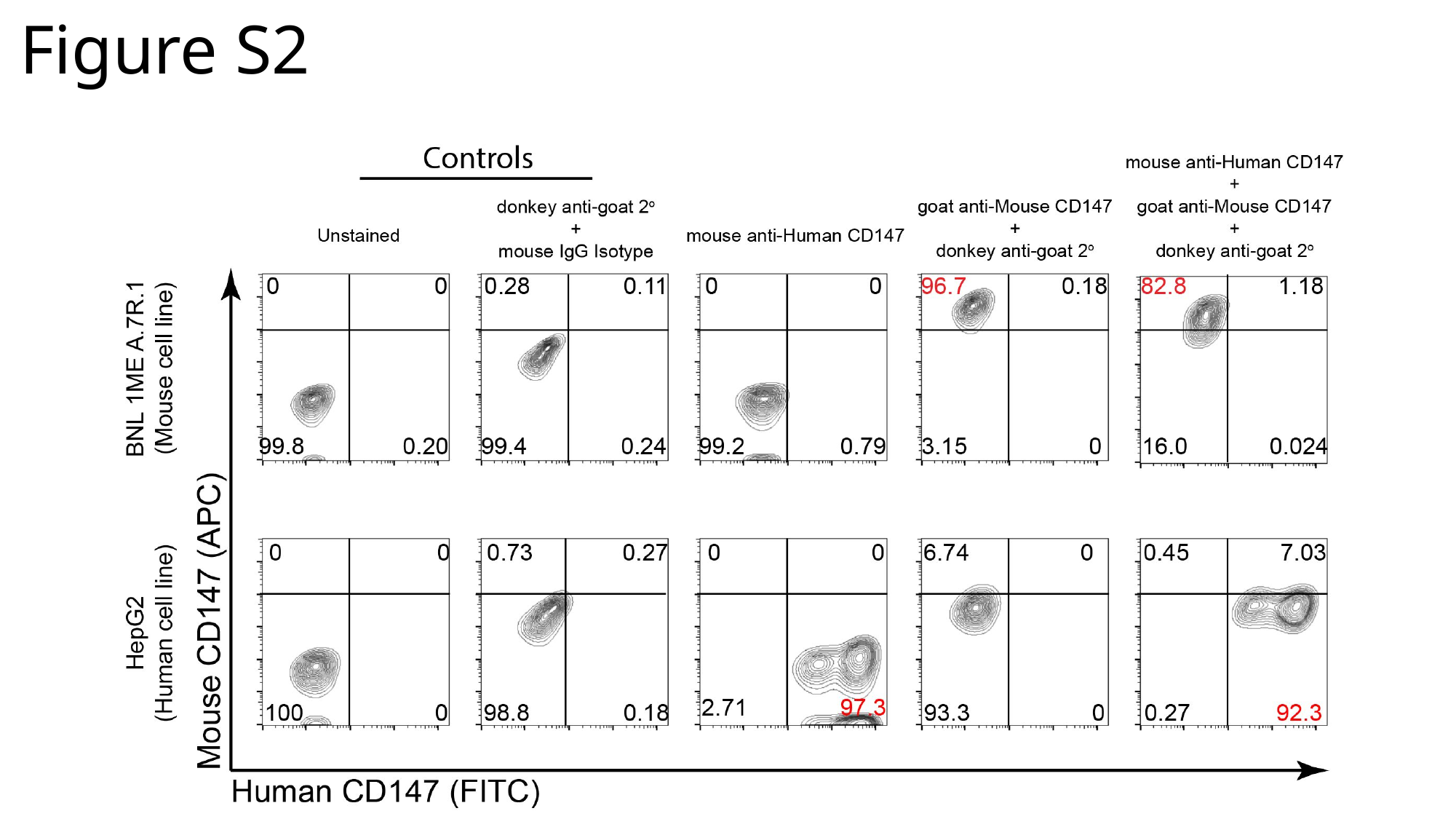

# Figure S2

## Slide 3
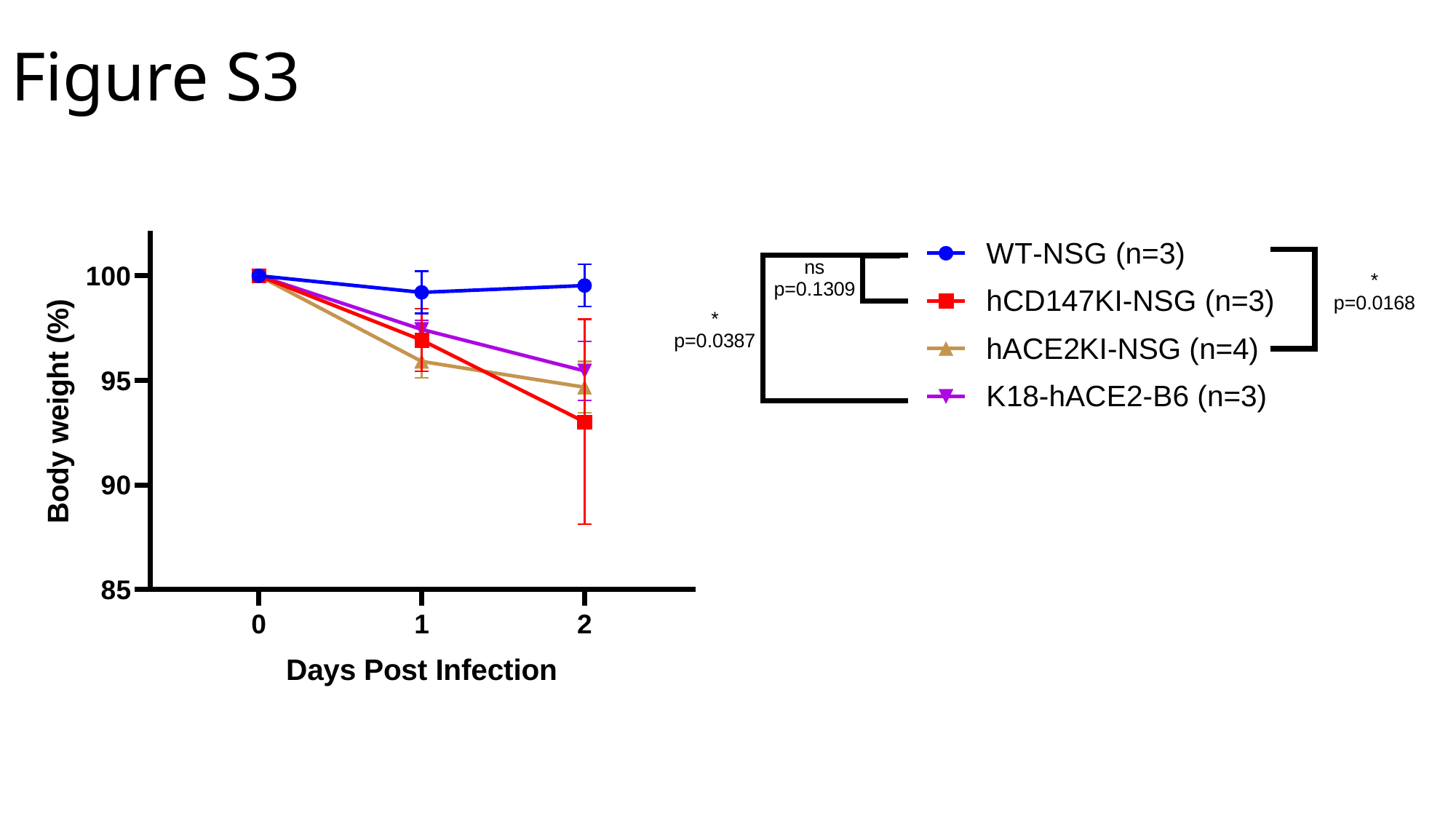

# Figure S3

## Slide 4
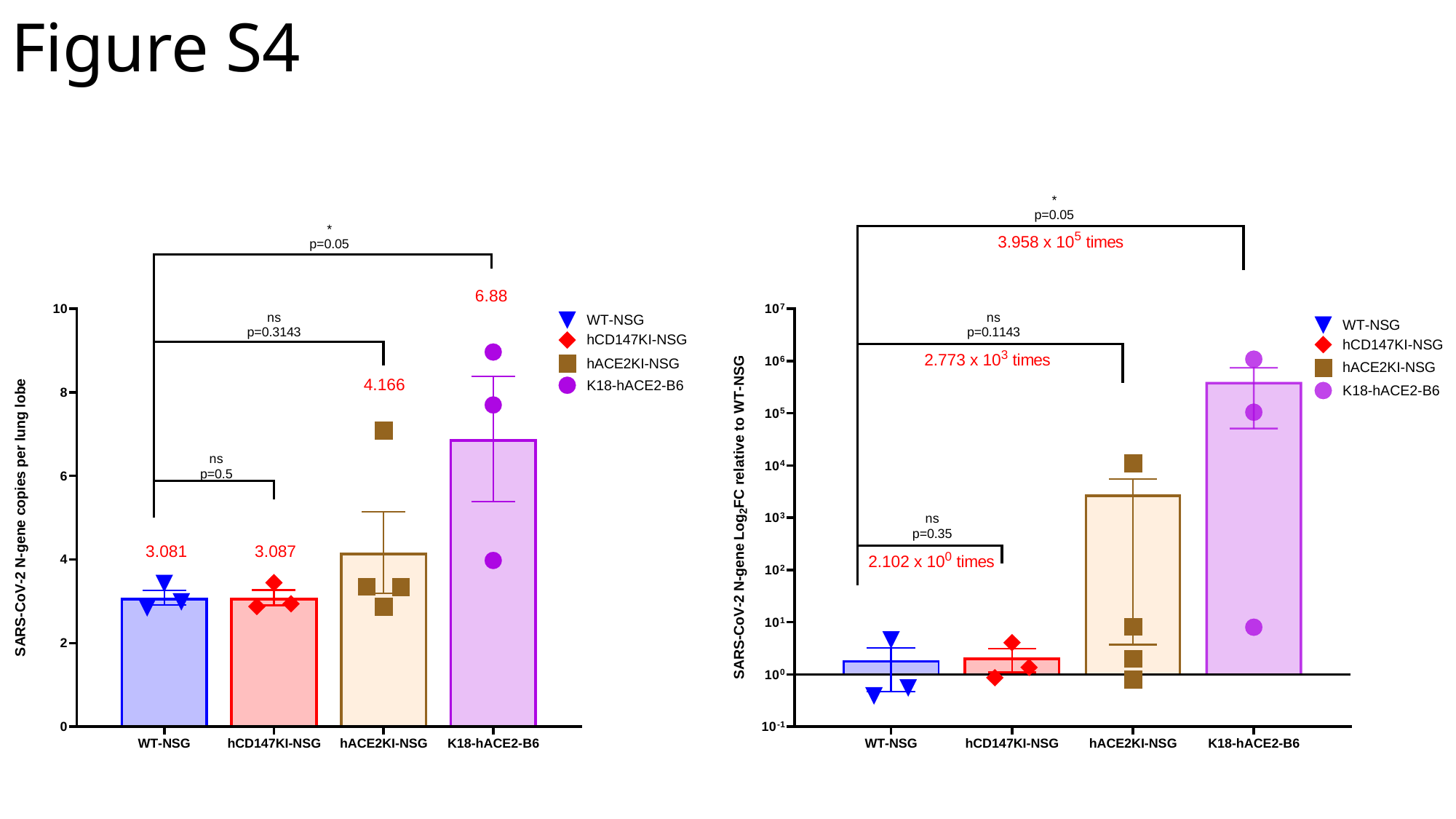

# Figure S4

## Slide 5
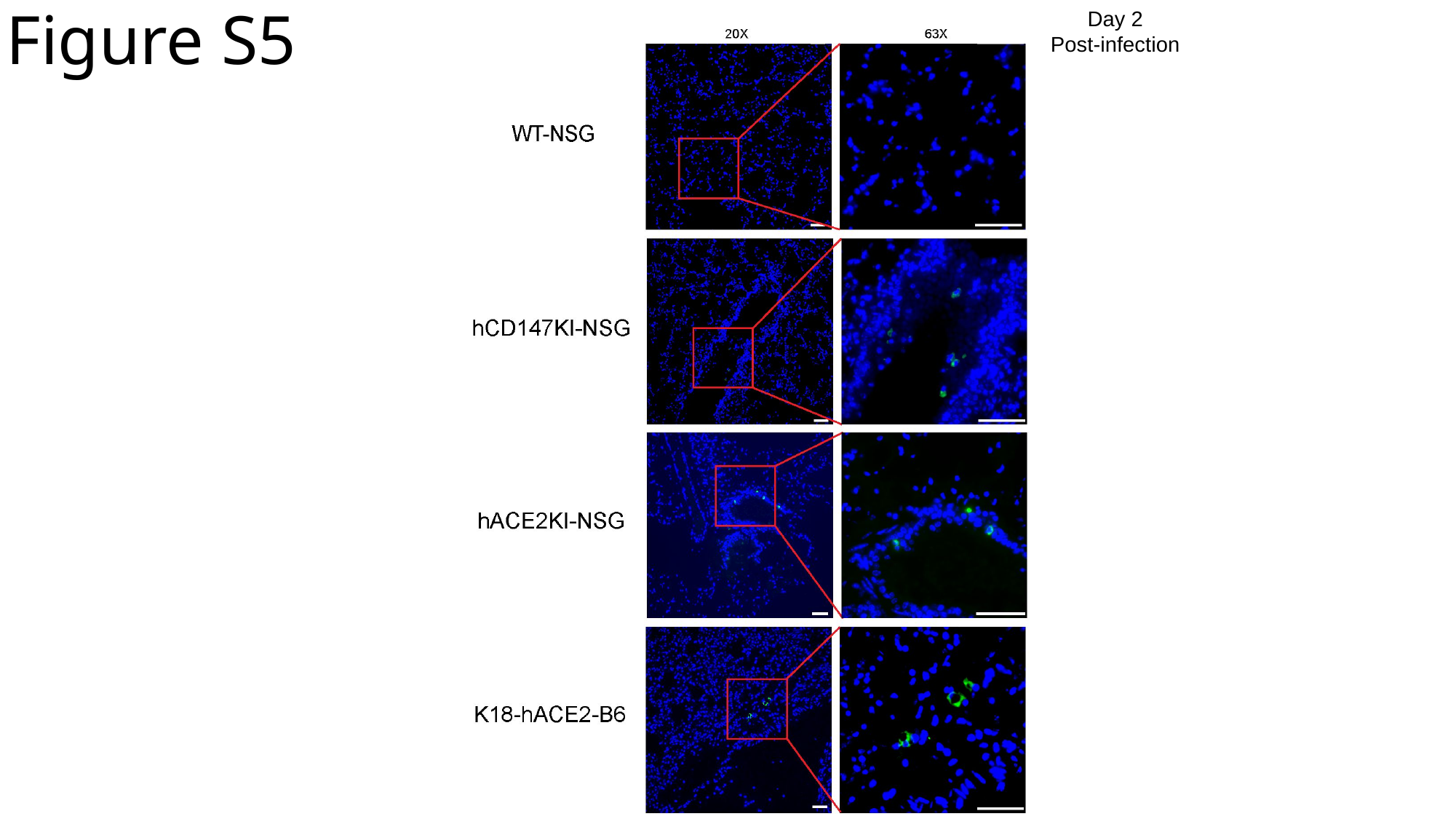

Day 2
Post-infection
# Figure S5

## Slide 6
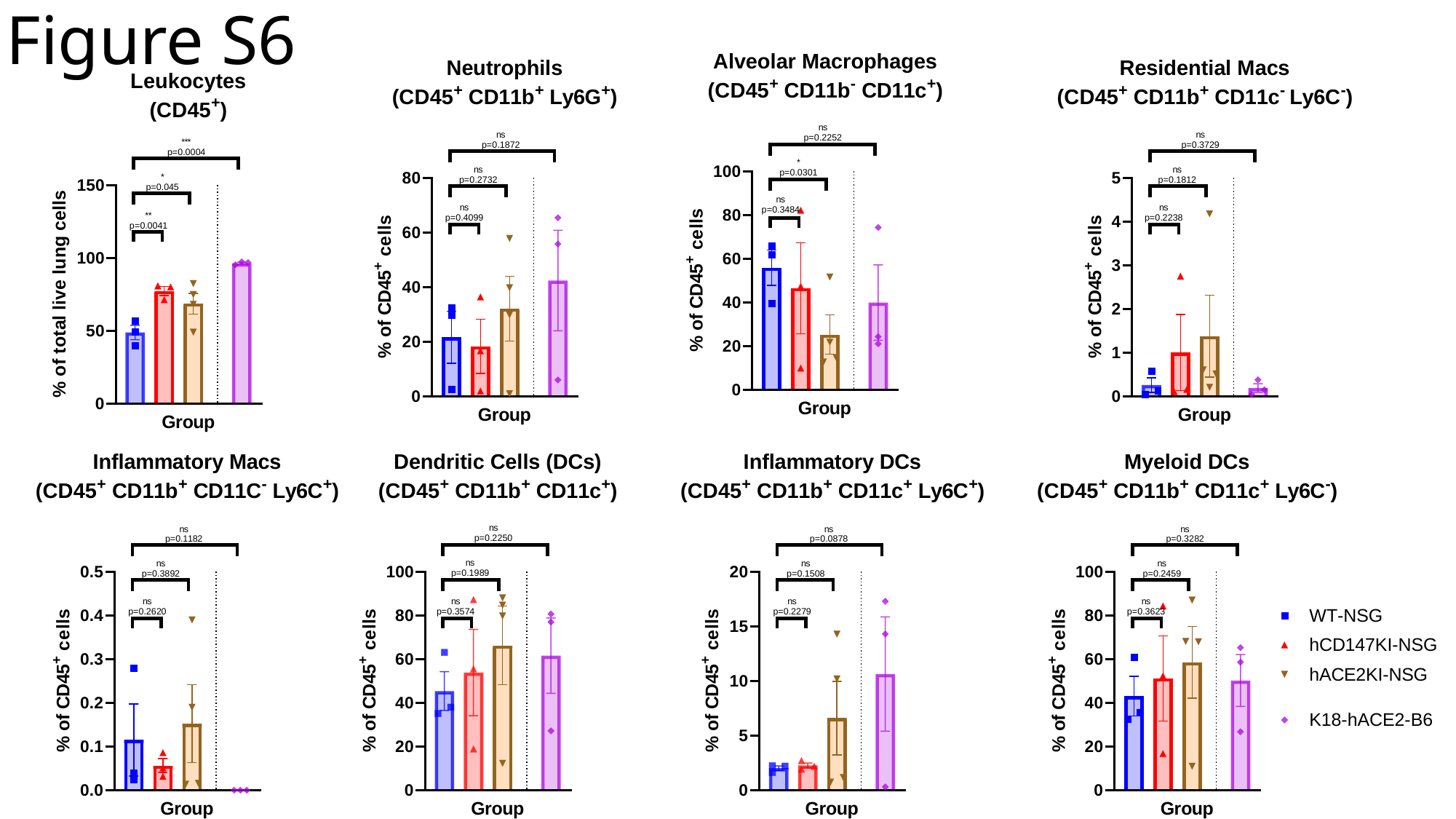

# Figure S6

## Slide 7
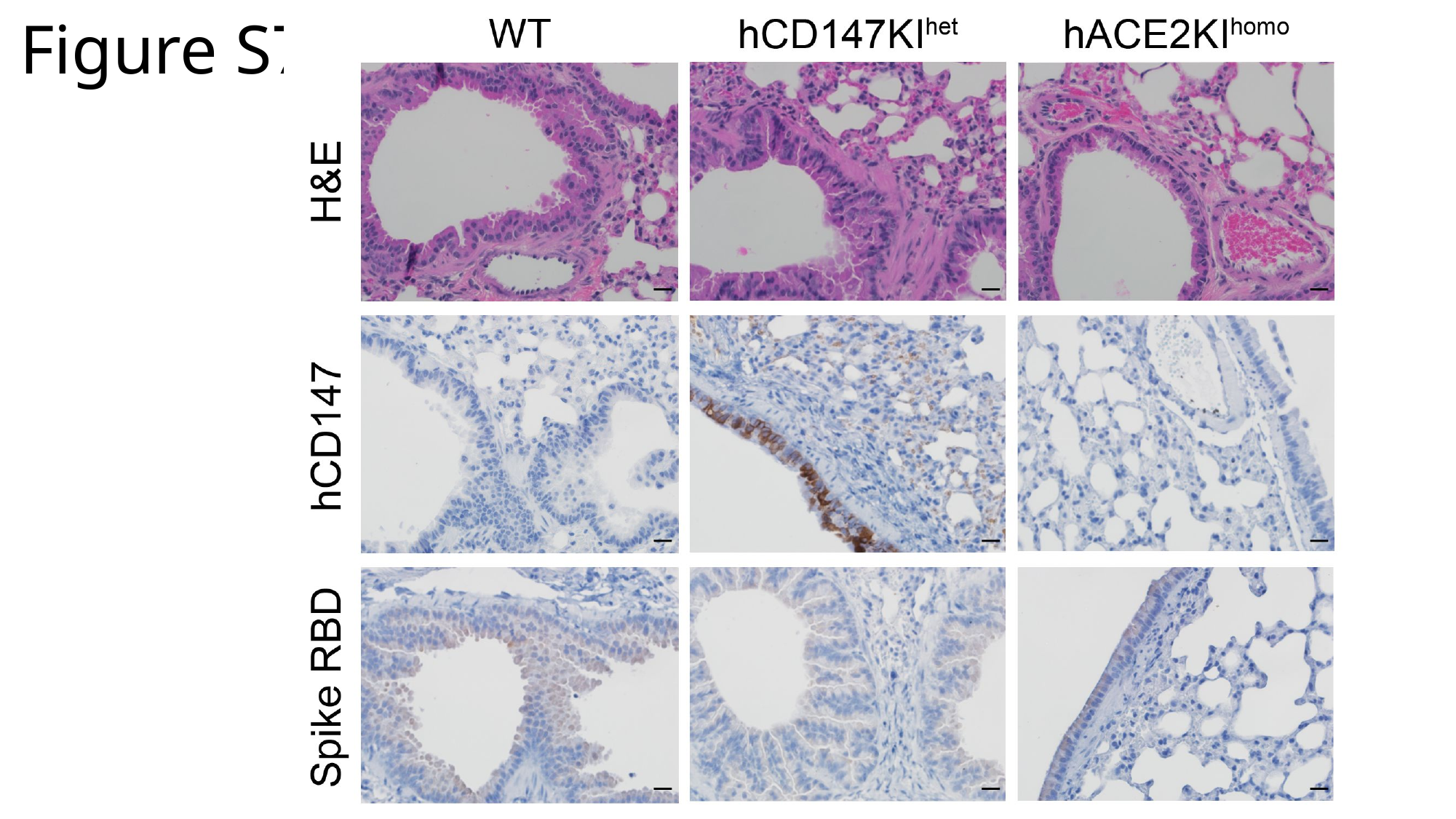

Figure S7

## Slide 8
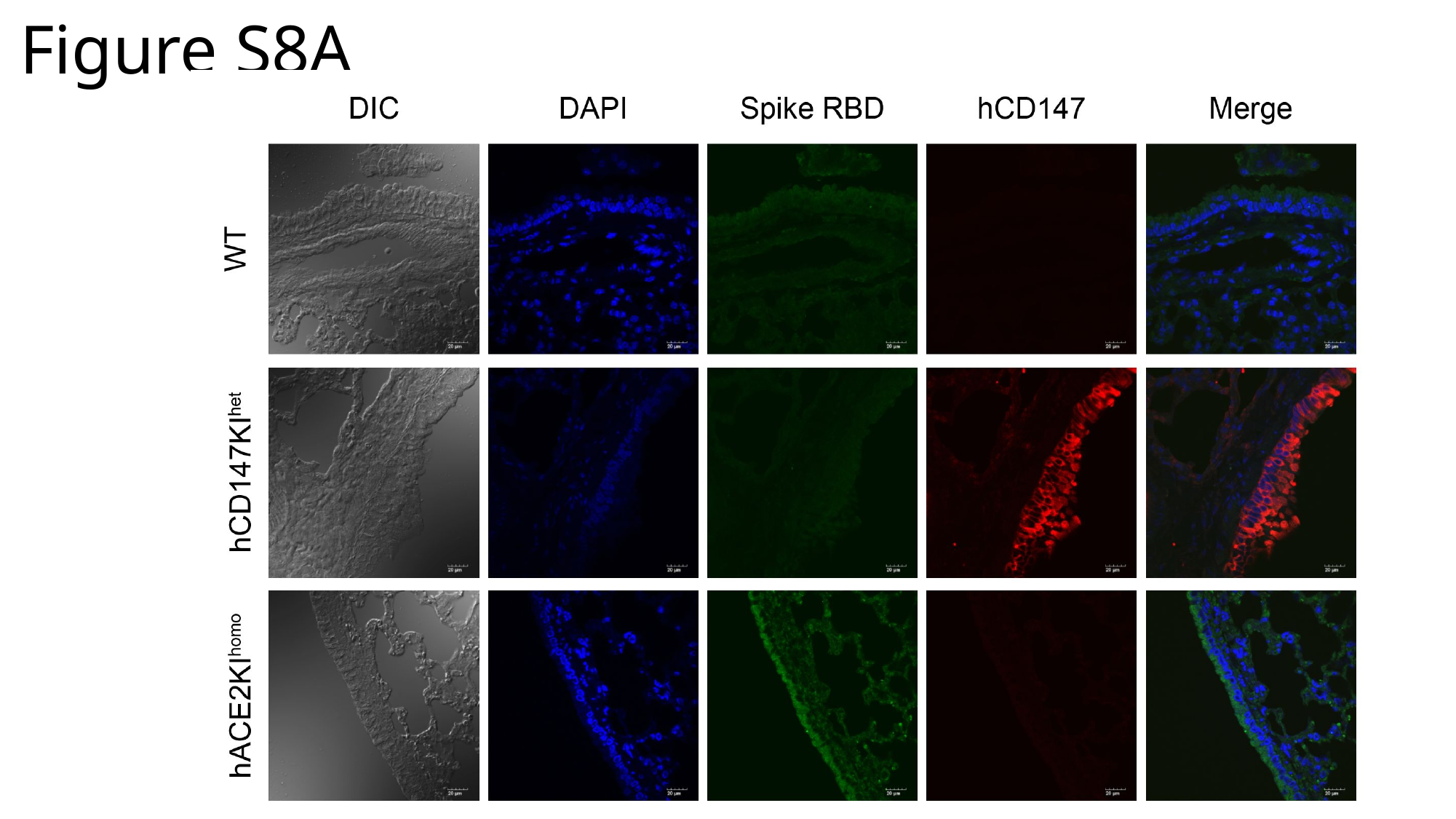

Figure S8A

## Slide 9
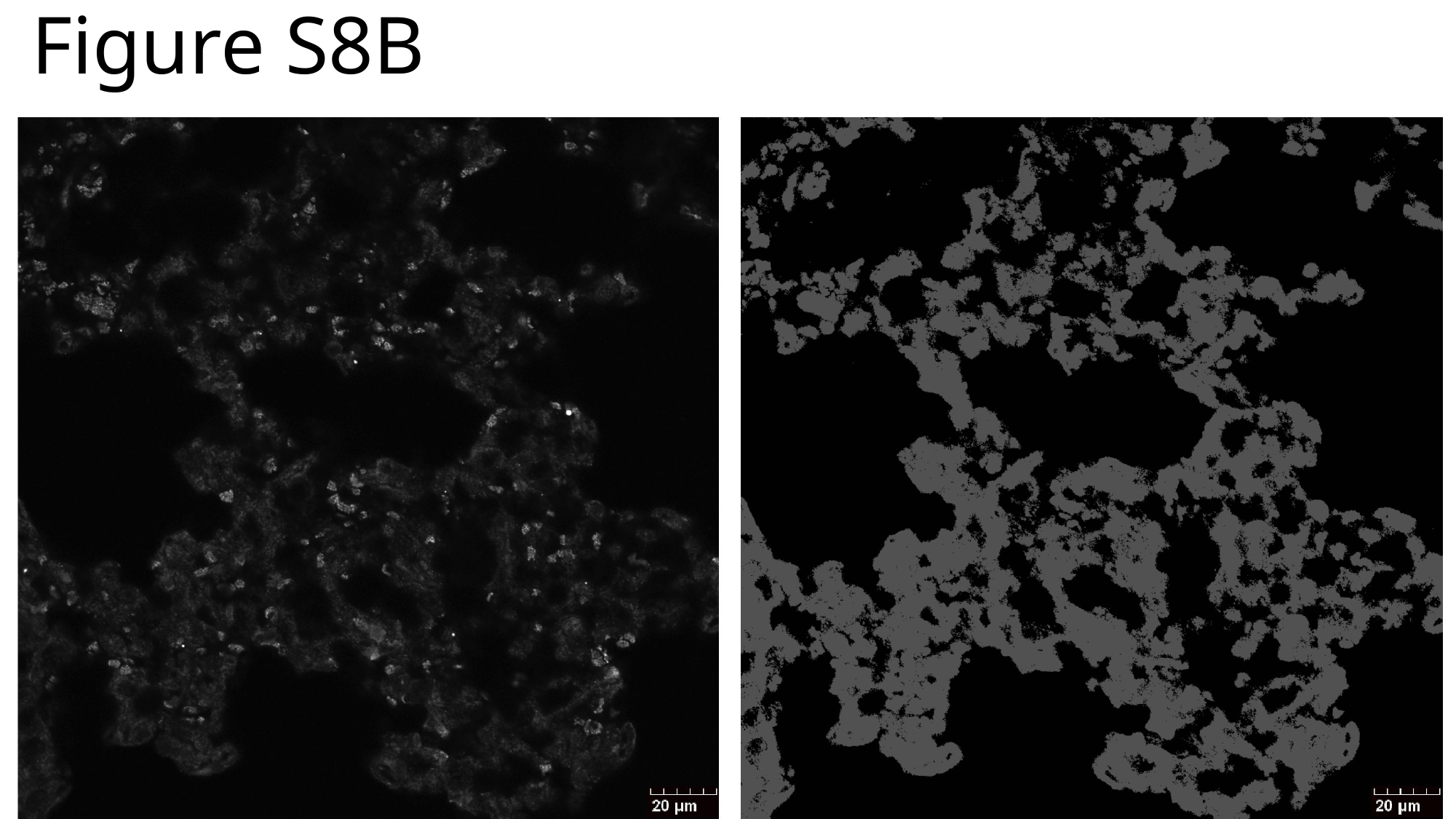

Figure S8B

## Slide 10
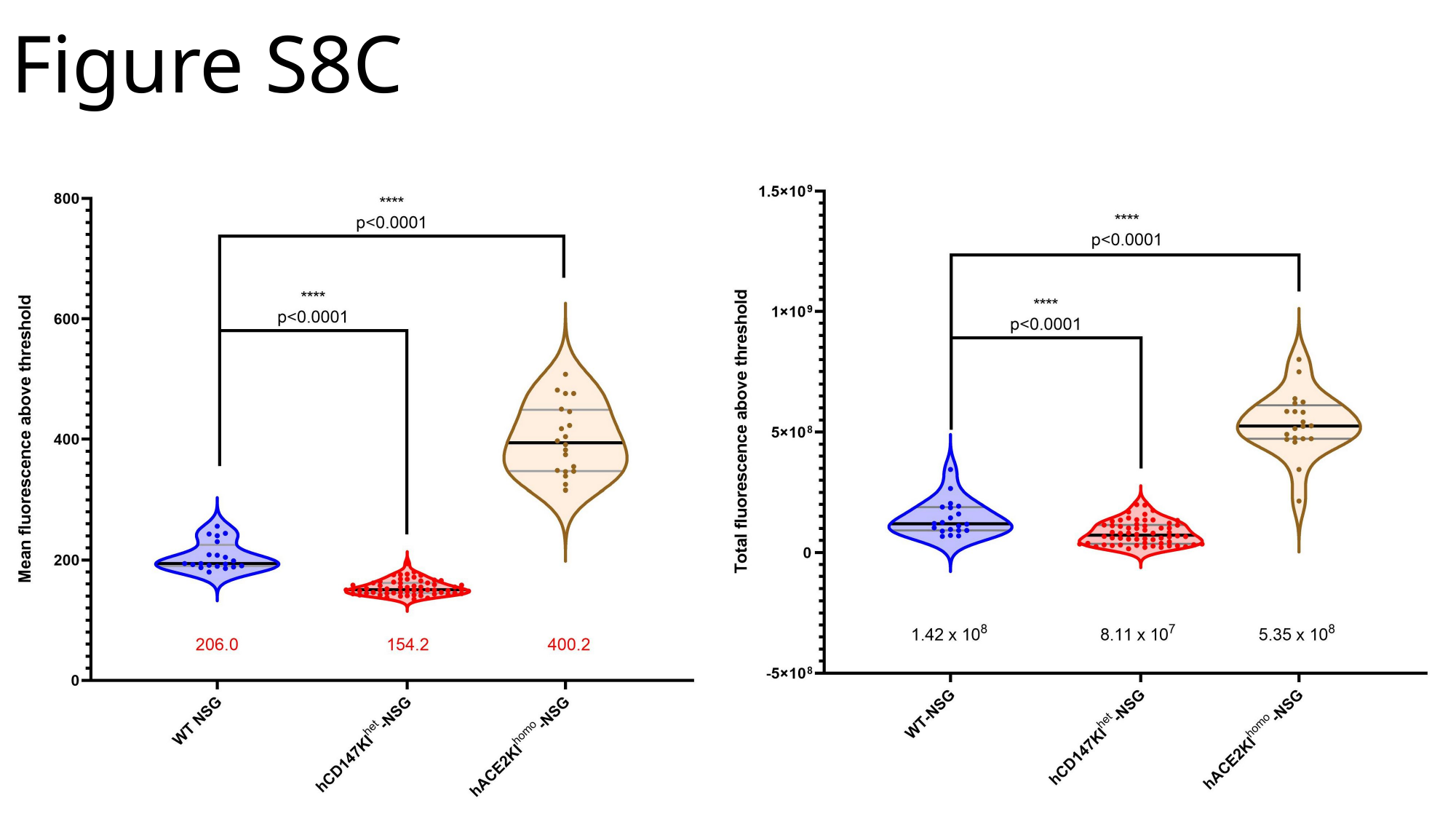

# Figure S8C

## Slide 11
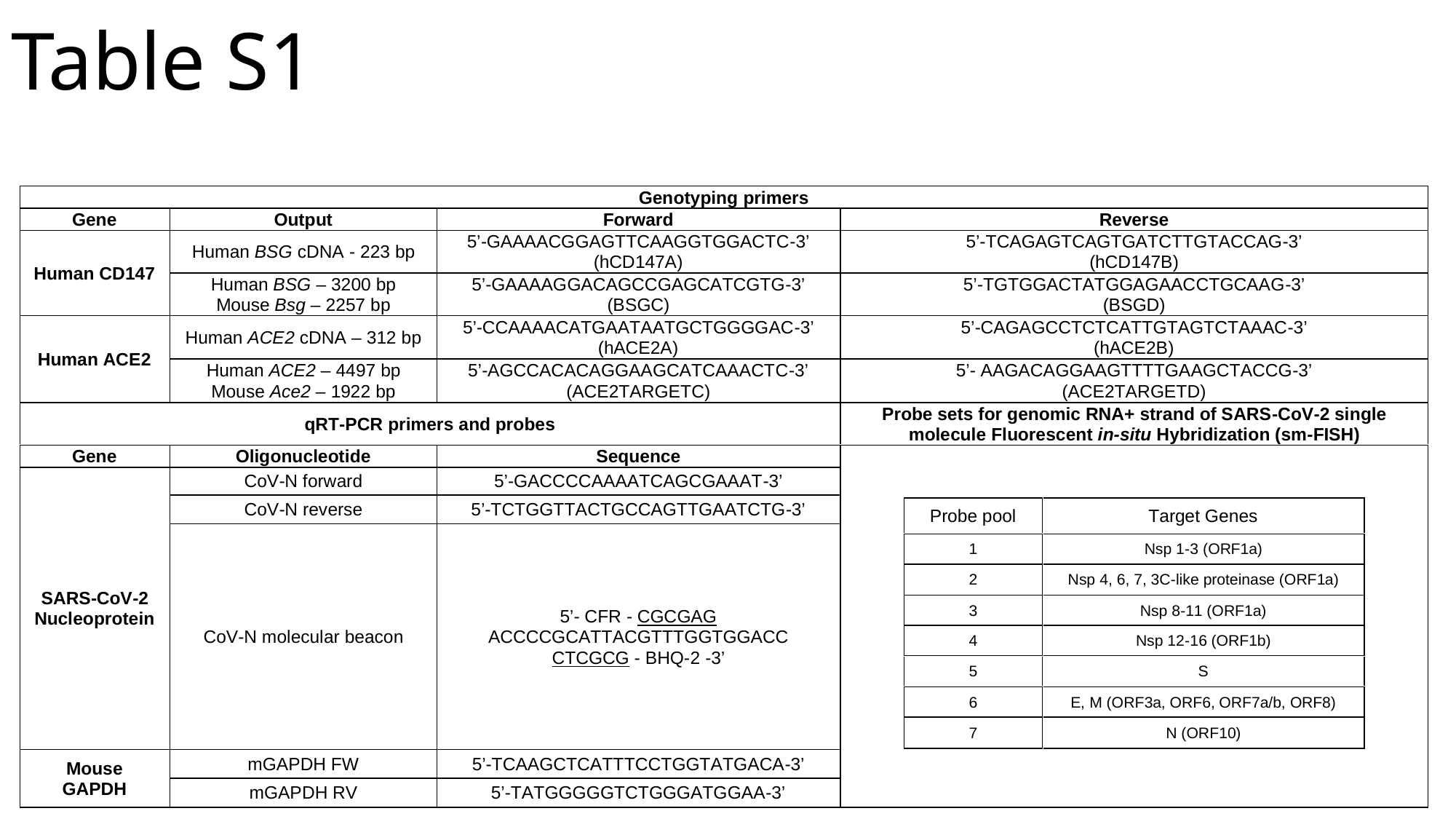

# Table S1
